# Dissecting the Ecological Structure of Health and Disease in the Global Gut Microbiome

**DOI:** 10.1002/advs.202517087

**Published:** 2026-05-19

**Authors:** Baoyuan Zhu, Shuhao Chen, Yunheng Diao, Wei Wang, Yuanyuan Huang, Liqin Liang, Xiaodan Lu, Rui Han, Minxin Guo, Zhaobo Li, Shuhong Wang, Hehua Li, Chenyu Liu, Jing Zhou, Dongsheng Xiong, Xiaobo Li, Yuping Ning, Xuetao Shi, Fengchun Wu, Kai Wu

**Affiliations:** ^1^ School of Materials Science and Engineering South China University of Technology Guangzhou China; ^2^ School of Biomedical Sciences and Engineering South China University of Technology Guangzhou International Campus Guangzhou China; ^3^ Department of Psychiatry The Affiliated Brain Hospital Guangzhou Medical University Guangzhou China; ^4^ Guangdong Engineering Technology Research Center for Translational Medicine of Mental Disorders Guangzhou China; ^5^ National Engineering Research Center for Tissue Restoration and Reconstruction South China University of Technology Guangzhou China; ^6^ Department of Biomedical Engineering New Jersey Institute of Technology Newark New Jersey USA; ^7^ Key Laboratory of Neurogenetics and Channelopathies of Guangdong Province and the Ministry of Education of China Guangzhou Medical University Guangzhou China; ^8^ Department of Aging Research and Geriatric Medicine Institute of Development Aging and Cancer Tohoku University Sendai Japan

**Keywords:** clinical association, ecological factor modeling, gut microbiota, major psychiatric disorders, non‐negative matrix factorization

## Abstract

The gut microbiota plays a crucial role in human health, but its coordinated ecological dynamics remain largely unclear. We present Wiredancer, a novel scalable framework based on similarity‐constrained non‐negative matrix factorization (NMF), which extracts continuous and overlapping microbial ecological factors (MEFs). By integrating 20,178 metagenomes spanning 36 countries and over 50 disease states, Wiredancer identified three robust and interpretable MEFs delineating the health‐disease continuum. MEF1, the dysbiotic factor dominated by *Bacteroides uniformis*, was elevated in disease populations; MEF2, the protective factor characterized by *Prevotella copri*, was reduced compared with the healthy group; and MEF3, the intermediate factor represented by *Bifidobacterium adolescentis*, reflected a mixed ecological configuration between MEF1 and MEF2. MEFs exhibited high reproducibility across individuals and longitudinal cohorts, but showed significantly increased variability in disease, consistent with the Anna Karenina principle and highlighting disrupted ecological stability. These findings were validated in the largest Chinese metagenomic cohort of major psychiatric disorders, where MEFs were associated with clinical symptoms, peripheral biomarkers, and disease subtypes, and remained essentially stable under short‐term treatment. Together, Wiredancer provides a generalizable strategy to define microbiome states and decode ecological transitions, offering new opportunities for precision diagnostics and stratified medicine in complex disorders.

## Graphical abstract

We introduce Wiredancer, a framework that identifies three continuous ecological factors of the gut microbiota. These factors exhibit distinct patterns across health and disease, jointly capturing disrupted ecological stability and offering a new perspective for precision diagnostics and therapeutic strategies.

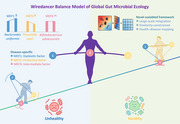

## 1 Introduction

The gut microbiota is crucial in sustaining host health [1, 2], and its stability is influenced by genetic predisposition and environmental exposures [3, 4, 5, 6]. In recent years, there has been growing evidence that gut microbiota dysbiosis may be involved in the pathogenesis of various diseases [7, 8, 9], especially major psychiatric disorders [10, 11, 12] such as major depressive disorder (MDD), schizophrenia (SZ), and bipolar disorder (BD). Although these disorders affect millions worldwide and impose a growing public health burden, their diagnosis remains primarily symptom‐based and lacks objective biomarkers [13, 14]. This gap underscores the necessity for approaches that can link gut microbial alterations to clinical phenotypes.

With growing interest in microbiota‐disease associations, numerous studies have reported significant alterations in gut microbial diversity and disruptions in the core gut microbiota in a variety of disease states [15, 16, 17, 18, 19, 20, 21]. However, considerable heterogeneity has been observed across studies [22], likely reflecting differences in cohort composition, sample size, data preprocessing, and analytical methods [23]. This inconsistency reflects the lack of large‐scale, globally representative microbiome landscapes processed with standardized pipelines, as well as the absence of approaches capable of capturing the ecological continuum and dynamic perturbations associated with disease. At the methodological level, traditional case‐control analyses can effectively explain group‐level differences in microbial features, but new frameworks are still needed to characterize the coordinated dynamics of the gut microbiota as an integrated ecosystem [24]. Moreover, given the substantial variability in individual health status, robust measures to characterize the dynamic changes in individual gut microbial composition are still lacking [25, 26]. Together, these limitations underscore the need for novel analytical frameworks that reconcile individual‐level variability with group‐level coordination in the gut microbiome.

The classic enterotype model has provided important insights into population heterogeneity and established a foundational framework for classifying gut microbial community structures [27]. Studies based on partitioning around the medoid [28] (PAM) and Dirichlet multinomial mixture models [29] (DMM) have identified three to four major enterotypes and demonstrated strong robustness in bacterial and fungal community analysis [30]. Nevertheless, these discrete classification approaches still involve a certain degree of simplification in capturing the complexity of microbiota variation, as reflected by their limited ability to represent the continuity and dynamic nature of microbial community states [31]. Drawing on economic physics and complex systems theory, we aim to construct a set of microbial ecological factors (MEFs) that capture the continuous variation of microbial communities and collectively define the latent ecological signature underlying individual health phenotypes. This idea is similar to the application of factor analytic methods, such as latent Dirichlet allocation to reveal structural and functional heterogeneity of the brain in MRI studies [32]. Although the data modalities are different, both face the need to extract low‐dimensional latent factors from high‐dimensional complex patterns. In addition, non‐negative matrix factorization (NMF) has been applied to microbiome data, supporting its utility for capturing latent ecological structures [33]. NMF‐derived latent factors can be viewed as continuous and partially overlapping ecological axes capturing coordinated variation in microbial community structure, forming a biologically interpretable low‐dimensional representation. However, current approaches still require improved high‐resolution characterization of microbial community structures [34]. Moreover, growing attention has been directed toward microbial structural similarity, given its importance for systematically delineating healthy microbiome states [35]. Improving species‐level resolution and jointly considering inter‐individual heterogeneity and intra‐individual similarity may help to more comprehensively characterize the biological features and potential functional roles of the gut microbiota.

At the same time, the health‐disease continuum paradigm has attracted increasing attention, challenging the traditional binary model. While binary comparisons remain useful for detecting group‐level differences, health and disease are better understood as positions along a continuous spectrum, with disease representing a deviation from the optimal state of health. Moreover, the Research Domain Criteria framework proposed by the National Institute of Mental Health [36], as well as the emerging concept of “p‐factors” in psychiatry [37], advocate a dimensional modeling approach to more closely match the complex phenotypic spectrum observed in clinical practice. Furthermore, from an ecosystem perspective, the gut microbiota constitutes an intricate system of diverse species and multilayered interactions, with microbial similarity requiring quantitative evaluation [38]. However, existing research has mainly focused on single species or local characteristics, and further efforts are needed to enable systematic analysis of the overall structure and dynamic coordination of microbial communities. Although a limited number of studies have explored the dynamic interplay between the gut microbiota and host physiological states and provided initial insights [39], there remains a need to establish quantitative frameworks to characterize how microbial communities change and interact during disease‐related state transitions. A key unresolved issue is how to systematically define the healthy homeostasis of the gut microbiota and track its changing trajectory during the transition to the host state.

**FIGURE 1:**
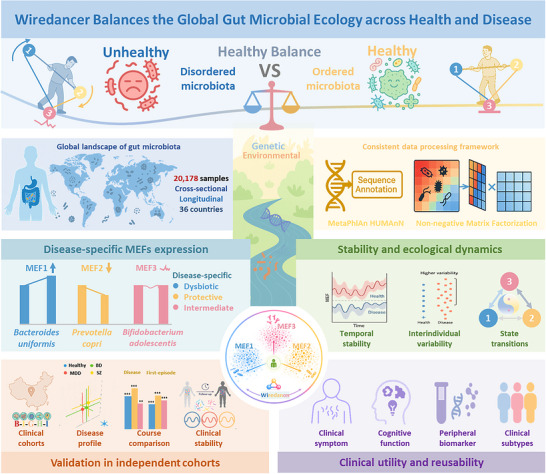
Schematic overview of the Wiredancer framework for MEFs modeling and validation. Global metagenomic sequencing data were first integrated and processed to generate microbial composition matrices, which served as the input for factor modeling. Through optimized NMF, three stable and biologically interpretable MEFs were identified. Each sample was then projected into a latent factor space, enabling the characterization of individual‐level ecological compositions and their associations with disease states. To assess robustness, the stability of MEFs was systematically evaluated across multiple dimensions, including longitudinal temporal dynamics, inter‐individual variability, and ecological interactions among factors. This analysis revealed asymmetric transition patterns that reflect the dynamic balance of microbial ecosystems. Finally, the framework was applied to large cohorts of patients with major psychiatric disorders to demonstrate clinical utility in disease differentiation, subtype stratification, phenotype correlation, and longitudinal follow‐up.

In this study, we established Wiredancer, a modeling framework built on large‐scale metagenomic data that incorporates similarity‐constrained NMF to capture continuous and overlapping MEFs (Figure 1). The framework is designed to balance ecological constraints with modeling flexibility, thereby enabling robust and interpretable characterization of complex microbiome states. Leveraging a globally collected and taxonomically diverse cohort of 20,178 samples, Wiredancer enables the systematic characterization of gut microbiota states across health and multiple disease conditions. Importantly, Wiredancer defines a stable ecological reference space where disease states are represented as structured deviations from a shared microbial system, enabling consistent cross‐cohort representation. Furthermore, validation in the largest cohort of patients with major psychiatric disorders demonstrated its potential to link microbial ecological patterns with clinical phenotypes. Overall, Wiredancer provides a scalable and generalizable strategy for capturing the ecological organization of the gut microbiota and linking it to clinical phenotypes, thereby advancing microbiome‐based diagnostics in complex diseases.

## 2 Results

### 2.1 Microbial Characteristics of the Participants

**FIGURE 2:**
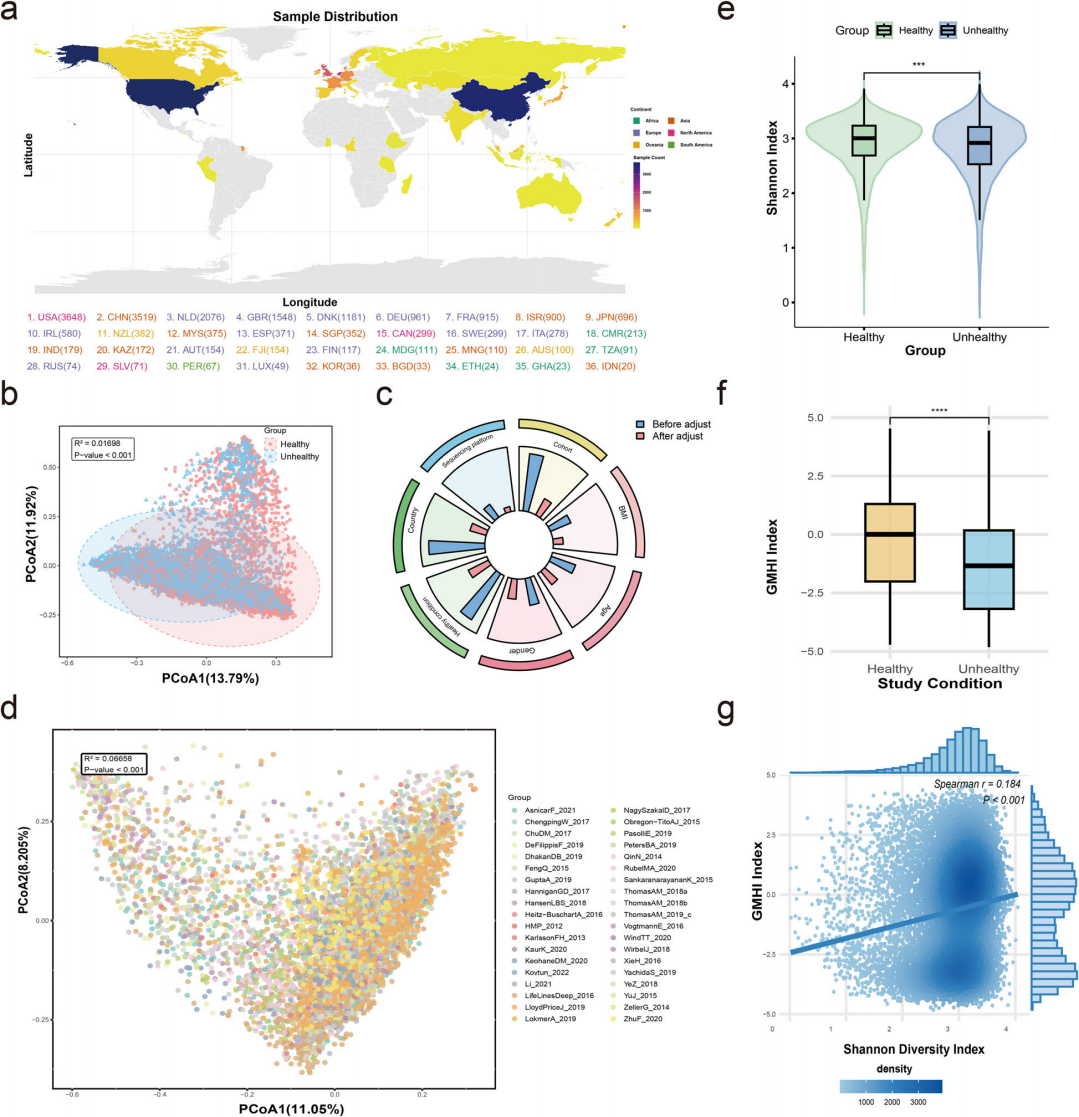
Integrated analysis of large‐scale metagenomic data reveals gut microbiota differences associated with health status. (a) Global distribution of 20,178 human gut metagenomic samples from 36 countries. Countries are colored by sample count. Map generated by the authors using R (sf, ggplot2, and rnaturalearth packages) based on publicly available geographic boundary data from Natural Earth (public domain). Country abbreviations are shown using ISO 3166‐1 alpha‐3 codes. This map is intended solely to illustrate sample distribution, and the boundaries and names shown do not imply any official endorsement or acceptance. (b) Principal coordinates analysis (PCoA) based on Bray–Curtis dissimilarity shows significant β‐diversity differences between healthy and unhealthy groups, as assessed by permutational multivariate analysis of variance (PERMANOVA, *p* < 0.001). (c) Circular plot illustrates cohort‐wise compositional structure before and after batch adjustment. (d) PCoA plot colored by study group after batch effect correction, indicating retained biological variation. (e) Violin and box plots showing Shannon diversity index significantly higher in the healthy group (Wilcoxon rank‐sum test, two‐sided; *n* = 20,178; ***, *p* < 0.001). (f) Box plot of GMHI, showing significantly greater values in healthy individuals (Wilcoxon rank‐sum test, two‐sided; *n* = 20,178; ****, *p* < 0.0001). (g) Spearman correlation between Shannon index and GMHI (*n* = 20,178; *r* = 0.184, *p* < 0.001) with density overlay.

We collected a global metagenomic dataset comprising 20,178 samples collected across 36 countries (Figure 2a, Table S1). The cohort spans a wide spectrum of health conditions, including more than 50 previously reported diseases and healthy states, providing a globally representative landscape of gut microbial features. For subsequent analyses, we selected samples with complete metadata on sex, age, body mass index (BMI), and sequencing platform, to ensure robust assessment of host and technical factors influencing the gut microbiota. Building on the microbial Anna Karenina principle and the framework of normative modeling, we used the healthy population as a relatively stable reference distribution to characterize background variability and identify systematic deviations from this baseline. Initial analyses revealed significant differences in the compositional structure (*p* < 0.001) of the gut microbiota between healthy and unhealthy groups (Figure 2b). However, because such differences could be confounded by batch effects across cohorts, we applied covariate adjustment using the MMUPHin framework (Figure S1a). After removing the effects of confounding variables, the microbial structural differences were markedly attenuated (Figure 2c, Table S2), with batch effects substantially reduced (Figure 2d).

We applied the MaAsLin2 framework to characterize microbial differences between the healthy and unhealthy groups, identifying 190 significant microbial features after multiple testing correction (Figure S2, Table S3). For instance, *Bacteroides stercoris*, *Streptococcus gordonii*, and *Clostridium symbiosum* were significantly increased in the unhealthy group, whereas *Prevotella copri*, *Roseburia inulinivorans*, and *Faecalibacterium prausnitzii* were significantly decreased compared to the healthy individuals. In addition, both the Shannon and Simpson indices were significantly changed in the unhealthy group (*p* < 0.001), although the magnitude of difference was relatively modest (Figure 2e; Figure S1b, Table S4). At the subgroup level of healthy group, Shannon diversity was significantly lower in males than females (*p* = 1.4 × 10^−15^; Figure S1c, Table S5), slightly lower in younger adults (<60 years) compared to older adults (≥60 years) (*p* = 0.0036; Figure S1d), and showed no significant difference across BMI groups (*p* = 0.35; Figure S1e). Additionally, we observed a significant decrease in the Gut Microbiota Health Index (GMHI) in the unhealthy group (*p* < 0.0001) compared to healthy individuals (Figure 2f). In addition, GMHI was also significantly positively correlated (*r* = 0.184, *p* < 0.001) with Shannon species diversity (Figure 2g). These findings suggest that gut microbial ecology reflects global health status at the group level. However, the relatively small effect size suggests that single ecological indices provide only a partial view and may not adequately resolve more subtle ecological or clinical heterogeneity, highlighting the need for additional complementary factors.

### 2.2 Species‐Level Analysis of Traditional Enterotype Models

**FIGURE 3:**
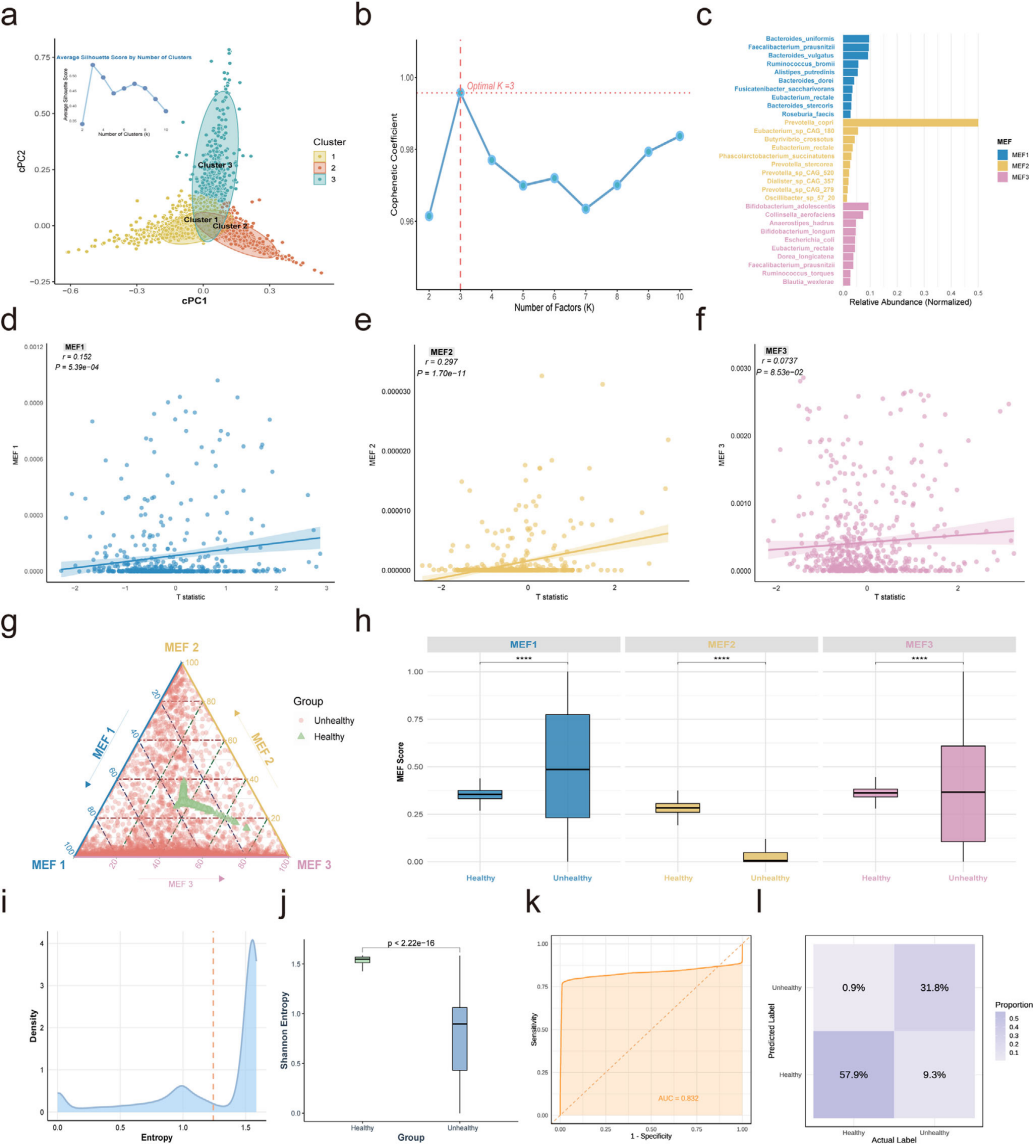
Identification and characterization of MEFs derived from Wiredancer. (a) Contrastive learning revealed three distinct health‐associated patterns among healthy samples. (b) Cophenetic correlation curve across varying factor numbers in Wiredancer, with optimal rank determined at *K* = 3. (c) Top 10 ranked taxa for each MEF, showing distinct compositional profiles. (d–f) Scatter plots show Pearson correlations between species‐level t‐statistics and taxon loadings of each MEF. (g) A ternary plot illustrates MEF compositions of individual samples, colored by health status, highlighting group‐level shifts in factor space between healthy and unhealthy groups. (h) Boxplots comparing MEFs scores between healthy and unhealthy groups, with all differences reaching significance (Wilcoxon rank‐sum test, two‐sided; *n* = 18,873; ****, *p* < 0.0001). (i) Density distribution of MEFs entropy showing a right‐skewed distribution. (j) MEFs' entropy significantly higher in healthy individuals. (k) ROC curve demonstrating the predictive performance of the factor‐based multivariable classification model using fivefold cross‐validation (AUC = 0.832). (l) Confusion matrix of the factor‐based classification model predicting health status based on fivefold cross‐validation, with an overall accuracy of 89.7% (*n* = 18,873).

Defining a healthy gut microbiota remains a fundamental question in microbiota research. Using a contrastive learning strategy, we uncovered three distinct and robust microbial modules in healthy individuals (Figure 3a), potentially reflecting stable species‐level compositional configurations associated with health. Additionally, we applied two classical clustering methods, including PAM and the DMM, to healthy individuals and extended their use to the species level in a large‐scale cohort, allowing for a more refined characterization of gut microbial community structures. In our large‐scale cohort of healthy individuals, PAM consistently identified two to three optimal clusters across various subsampling proportions, as supported by both the Calinski–Harabasz index and silhouette score (Figure S3a–c). Notably, PAM demonstrated high stability across repeated runs and was relatively robust to sample size variation. In contrast, the DMM model was markedly sensitive to sample size, with the optimal number of clusters increasing progressively as determined by the Akaike and Bayesian information criteria (Figure S4a–e). These results suggest that DMM exhibits unstable convergence with increasing sample size, reaffirming previous genus‐level observations at the species level. Despite their utility in delineating broad community types, these traditional clustering approaches exhibit inherent limitations. Discrete models such as PAM and DMM fail to capture the widespread mixed ecological features within individual microbiota, overlooking the continuum of microbial community states.

### 2.3 Identification of the MEFs via Wiredancer

Within the Wiredancer framework, each individual is represented by a continuous mixture of microbial factors, allowing the capture of both mixed community structures and ecological gradients, rather than being restricted to a single enterotype as in traditional clustering. Each latent factor corresponds to a microbial community pattern, while individual samples are represented as weighted combinations of multiple factors, reflecting the co‐existence and overlap of communities within the same host and capturing the continuous and complex nature of the gut microbiome. Increasing the number of factors improves the total explained variance, analogous to refining a color spectrum into narrower bands, but it also raises the risk of overfitting. By evaluating model stability using the cophenetic correlation coefficient, we determined that three factors (*K* = 3) consistently achieved the highest stability across different parameter settings and yielded consistent results under cohort‐stratified cross‐validation (Figure 3b; Figure S5, Table S6). The three MEFs were each dominated by distinct microbial communities—MEF1 by *Bacteroides uniformis, Faecalibacterium prausnitzii, Bacteroides vulgatus, Ruminococcus bromii, Alistipes putredinis*; MEF2 by *Prevotella copri, Butyrivibrio crossotus, Eubacterium rectale, Phascolarctobacterium succinatutens*; and MEF3 by *Bifidobacterium adolescentis, Collinsella aerofaciens, Anaerostipes hadrus* (Figure 3c, Table S7). We further observed strong interrelationships among distinct MEFs (Figure S6). At the functional level, the three MEFs also showed distinct metabolic profiles (Figure S7). MEF1 was enriched in carbohydrate and arginine metabolism, MEF2 in nucleotide and branched‐chain amino acid biosynthesis, and MEF3 in peptidoglycan maturation, together with carbohydrate fermentation and energy metabolism. Additionally, to evaluate the extent to which these factors capture disease‐associated global compositional variation, we developed an analysis framework based on global variation patterns. Specifically, we first computed species‐level t‐statistics comparing healthy and unhealthy cohorts to characterize the overall direction and magnitude of compositional shifts. We then assessed the correspondence between these global variation patterns (Figure S8) and the taxon loadings of each MEF using Pearson correlation. MEF1 showed a modest but significant association (*r* = 0.152, *p* = 5.39 × 10^−^^4^, Figure 3d), MEF2 showed a strong association (*r* = 0.297, *p* = 1.70 × 10^−^^1^ ^1^, Figure 3e), whereas MEF3 was only relatively weakly correlated (*r* = 0.0737, *p* = 8.53 × 10^−^^2^, not significant, Figure 3f), indicating that the first two factors predominantly underlie disease‑associated microbial variation at the global level.

We next examined differences in individual MEF expression between healthy and unhealthy groups, yielding striking results. Healthy subjects displayed tightly clustered factor scores, whereas unhealthy individuals exhibited markedly dispersed profiles (Figure 3g), consistent with the Anna Karenina principle. We then compared mean MEF scores between groups (Figure 3h) and found that MEF1 was significantly higher in the unhealthy group (*p *< 1 × 10^−^^4^), while MEF2 was significantly lower compared to healthy individuals (*p* < 1 × 10^−^^4^). Interestingly, although the median of MEF3 did not differ substantially between groups, unhealthy subjects showed greater variability in MEF3 expression (*p *< 1 × 10^−^^4^). Importantly, after stratifying individuals by disease states, this pattern was observed across most of the 51 distinct health conditions (Figure S9). We further examined the variations of the three MEFs across sex, age, and BMI groups. In the stratified analyses, only MEF2 showed a statistically significant difference across sex groups, whereas no significant differences were observed for MEF1 and MEF3 across sex, age, or BMI strata (Figure S10a–i). To quantify community heterogeneity, we computed the Shannon entropy of each sample's factor distribution, which was right‑skewed overall (Figure 3i) and significantly lower in the unhealthy group (Figure 3j). Finally, to assess the diagnostic utility of factor profiles, we built a logistic regression model with fivefold cross‑validation, with an area under the ROC curve reaching 0.832 (Figure 3k; Figure S11a) and an overall classification accuracy of 89.7% (Figure 3l). Additionally, we found that factor entropy enabled accurate classification of health status (Figure S11b).

### 2.4 MEFs Exhibit High Stability

**FIGURE 4:**
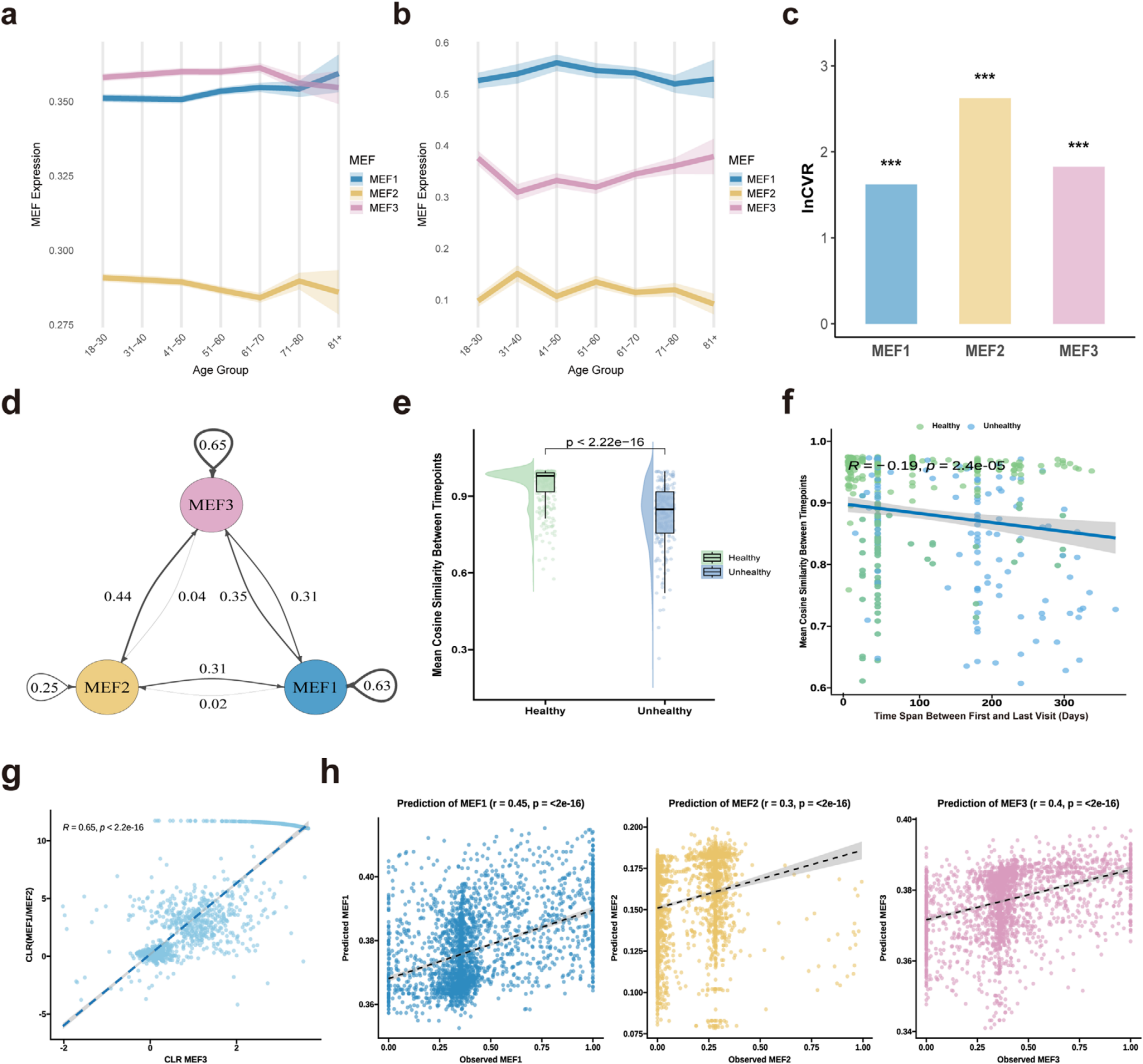
Analysis of MEFs stability and transitions. (a,b) Line plots show MEFs scores across different age groups in the healthy (a) and unhealthy (b) populations. (c) Bar plot illustrates significantly elevated lnCVR for all MEFs in the unhealthy group (permutation test; *n* = 18,873; ***, *p *< 0.001), suggesting greater inter‐individual variability. (d) The Markov chain transition network depicts the transitions among the three MEFs, with arrow direction indicating transition direction and thickness/values representing transition probabilities (*n* = 18,873). (e) Box and violin plots compare cosine similarity of MEFs compositions between follow‐up samples in healthy and unhealthy groups, showing significantly lower similarity in the unhealthy group (Wilcoxon rank‐sum test, two‐sided; *n* = 498; *p* < 2.2 × 10^−16^). (f) Linear regression shows that across all individuals, longer time intervals between visits are associated with lower MEFs similarity (*r* = −0.19, *p* = 2.4 × 10^−5^). (g) CLR‐transformed MEF1/MEF2 scores show a strong association with MEF3 (*r* = 0.65, *p *< 2.2 × 10^−16^), supporting the validity of MEFs inference. (h) Scatter plots illustrate the Spearman correlations between predicted and observed MEFs scores, with MEF1 (*r* = 0.45), MEF2 (*r* = 0.30), and MEF3 (*r* = 0.40) all showing robust performance (*n* = 498, all *p* < 2.2 × 10^−16^).

To evaluate the stability of MEFs derived from Wiredancer, we adopted a two‐stage analytical framework combining cross‐sectional and longitudinal data. In the cross‐sectional analysis, we first examined age‐related trajectories of MEFs expression in a globally diverse healthy population. MEF1 expression showed a slight age‐related increase, MEF2 declined steadily, and MEF3 followed an inverted U‐shaped trajectory with a peak between 60 and 70 years, suggesting a potential ecological inflection point in later life (Figure 4a). In contrast, the unhealthy group displayed greater fluctuations across all three MEFs (Figure 4b), indicating more pronounced instability and dysbiosis. Additionally, to quantify compositional variability, we compared the log coefficient of variation ratio (lnCVR) across groups. All three MEFs exhibited significantly higher lnCVR values in the unhealthy population than in the healthy group (permutation test, *p* < 0.001; Figure 4c, Table S8), reflecting greater ecological volatility under disease conditions. Notably, MEF2 displayed the greatest variability, suggesting a strong association with disease‐related ecological instability.

In the longitudinal analysis, we constructed a Markov transition network using MEF1‐3 as discrete ecological states (Figure 4d, Table S9). MEF1 and MEF3 exhibited relatively high self‐transition probabilities (0.63 and 0.65, respectively), indicating greater temporal persistence of these states. In contrast, MEF2 showed a lower self‐transition probability (0.25) and more frequent transitions toward MEF1 (0.31) and MEF3 (0.44). Transitions from MEF1 and MEF3 to MEF2 were infrequent, suggesting asymmetric transition dynamics among the three states. Notably, MEF3 exhibited the highest self‐transition probability and maintained moderate transition probabilities to both MEF1 and MEF2, suggesting that MEF3 may represent an intermediate or transitional ecological state between MEF1 and MEF2.

### 2.5 Stability and Dynamic Reorganization of MEFs

We compared the overall temporal stability of MEFs composition using cosine similarity across repeated timepoints. The temporal stability of samples from unhealthy individuals was significantly lower than that of the healthy group (*p* < 2.22 × 10^−16^; Figure 4e, Table S10), although overall similarity remained high. Importantly, stability showed a significant negative correlation with the time span between follow‐up visits (*r* = −0.19, *p* = 2.4 × 10^−5^; Figure 4f), indicating that ecological consistency degrades with increasing temporal distance.

Furthermore, we used a linear mixed‐effects model to examine the relationship between MEF3 and MEF1/MEF2. CLR‐transformed MEF3 was positively correlated with MEF1/MEF2 expression (*r* = 0.65, *p* < 2.2 × 10^−16^; Figure 4g), indicating that MEF3 is associated with variation shared across these factors. Finally, we further evaluated the temporal predictability of MEFs expression, a property of key relevance for understanding the dynamic behavior of microbial communities. Predicted values for MEF1, MEF2, and MEF3 were highly correlated with observed measurements (all *p* < 2 × 10^−16^; Figure 4h), indicating that these ecological factors exhibit strong temporal reproducibility and learnability. Together, these findings indicate dynamic reorganization of MEFs, with directional transitions among MEF1, MEF2, and MEF3 reshaping ecological balance.

### 2.6 Disorder‐Specific Microbial Factor Alterations and Cognitive Disruption

**FIGURE 5:**
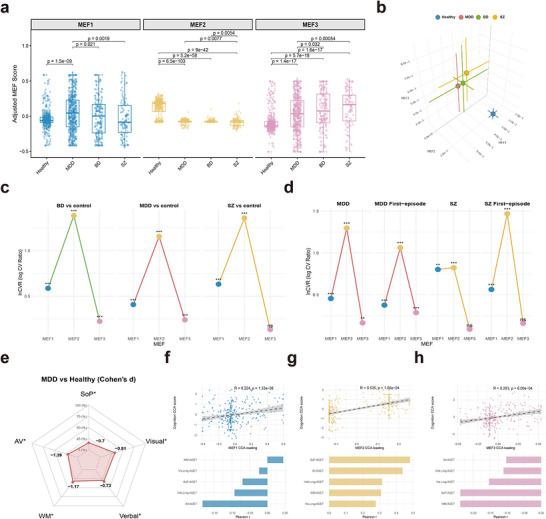
Disorder‐specific MEFs shifts and clinical relevance. (a) Boxplots display the distributions of MEF1‐3 scores across healthy, MDD, BD, and SZ groups (Wilcoxon rank‐sum test, two‐sided; *n* = 1,305). (b) 3D plot visualizes group‐level distributions in MEF space, highlighting directional deviations from the healthy reference. (c,d) lnCVR comparisons show increased inter‐individual variability in MEFs scores for psychiatric disorder groups compared to the healthy group (c), and between non‐first‐episode versus first‐episode subgroups relative to the healthy group (d) (permutation test; *n* = 1,305; **, *p* < 0.01; ***, *p* < 0.001). (e) Radar plot illustrates cognitive impairments in the MDD group compared to the healthy group across five domains, based on Cohen's *d* effect sizes (*n* = 402). (f–h) Canonical correlation analyses reveal associations between MEF1‐3 scores and cognitive performance. Scatter plots illustrate the CCA‐observed correlation with overall cognition, while bar plots display the Pearson correlation coefficients between MEFs and each cognitive domain (*n* = 471).

Using the Wiredancer framework, we integrated the publicly available metagenomic datasets with our independently established psychiatric cohort to systematically characterize the expression profiles of MEFs. Adjusted MEF expression differed significantly across diagnostic groups (Figure 5a, Table S11). Specifically, MEF1 was primarily increased in MDD; MEF2 was consistently decreased across MDD, BD, and SZ, exhibiting a shared cross‐disorder reduction pattern; in contrast, MEF3 was significantly increased across all three disorders, with more pronounced elevations in BD and SZ. When projected into three‐dimensional MEFs space, all three disorders exhibited directional shifts away from the healthy baseline (Figure 5b, Table S12). Although MDD, BD, and SZ are clinically classified as distinct disorders, they share overlapping symptoms. We found that SZ and BD were closest in spatial distribution, MDD also showed similarity to BD, while MDD and SZ were relatively more distant, closely mirroring their clinical symptom patterns. This observation challenges the traditional categorical model of microbiota dysregulation and supports a dimensional continuum model of ecological deviation from health.

We further assessed inter‐individual variability in MEFs expression across psychiatric disorder groups. Variability in MEF1 and MEF2 was significantly elevated in MDD, BD, and SZ (all *p* < 0.001), whereas increased MEF3 variability was observed only in MDD and BD, with no significant change detected in SZ (Figure 5c). Given that disease progression may affect gut microbial stability, we further compared first‐episode and non‐first‐episode patients within the MDD and SZ groups (Figure 5d). In MDD, both non‐first‐episode and first‐episode patients exhibited significantly greater MEFs variability compared to the healthy group (all *p* < 0.01). Notably, non‐first‐episode MDD patients showed higher variability in MEF1 and MEF2 than first‐episode patients, whereas MEF3 variability was lower. These findings suggest that the intermediate ecological pattern captured by MEF3 may become less prominent with disease progression, while instability in MEF1 and MEF2 intensifies. In contrast, MEF3 variability in SZ was not significantly altered in either non‐first‐episode or first‐episode patients compared to controls. However, non‐first‐episode SZ patients displayed increased MEF1 variability and decreased MEF2 variability relative to first‐episode patients, indicating diagnosis‐specific trajectories of microbial instability across psychiatric disorders. Together, these results demonstrate that the Wiredancer framework captures disorder‐specific alterations and progression trajectories that mirror the clinical heterogeneity of psychiatric disorders.

### 2.7 Associations Between MEFs and Cognitive Function

Given the well‐documented cognitive impairments in psychiatric populations, we next assessed performance across cognitive function (Figure 5e, Table S13). MDD patients exhibited significant deficits in speed of processing (SoP; Cohen's *d* = −0.7, *p *< 0.05), attention/vigilance (AV; Cohen's *d* = −1.39, *p *< 0.05), working memory (WM; Cohen's *d* = −1.17, *p *< 0.05), verbal learning (Cohen's *d* = −0.73, *p *< 0.05) and visual learning (Cohen's *d* = −0.81, *p *< 0.05). SZ patients also showed strong impairments (Figure S12), exemplified by deficits in attention/vigilance (Cohen's *d* = −0.73, *p *< 0.05) and verbal learning (Cohen's *d* = −0.72, *p *< 0.05). Furthermore, canonical correlation analysis (CCA) revealed significant associations between MEFs expression and cognitive function, with MEF1 and MEF3 showing predominantly negative correlations across cognitive function, whereas MEF2 was positively correlated with all cognitive measures (Figure 5f–h), and these associations remained significant after covariate adjustment (Figure S13). Thus, the ecological factors derived from the Wiredancer framework reveal a significant association between microbiome variation and cognitive phenotypes.

### 2.8 Clinical Validation of MEFs in MDD

**FIGURE 6:**
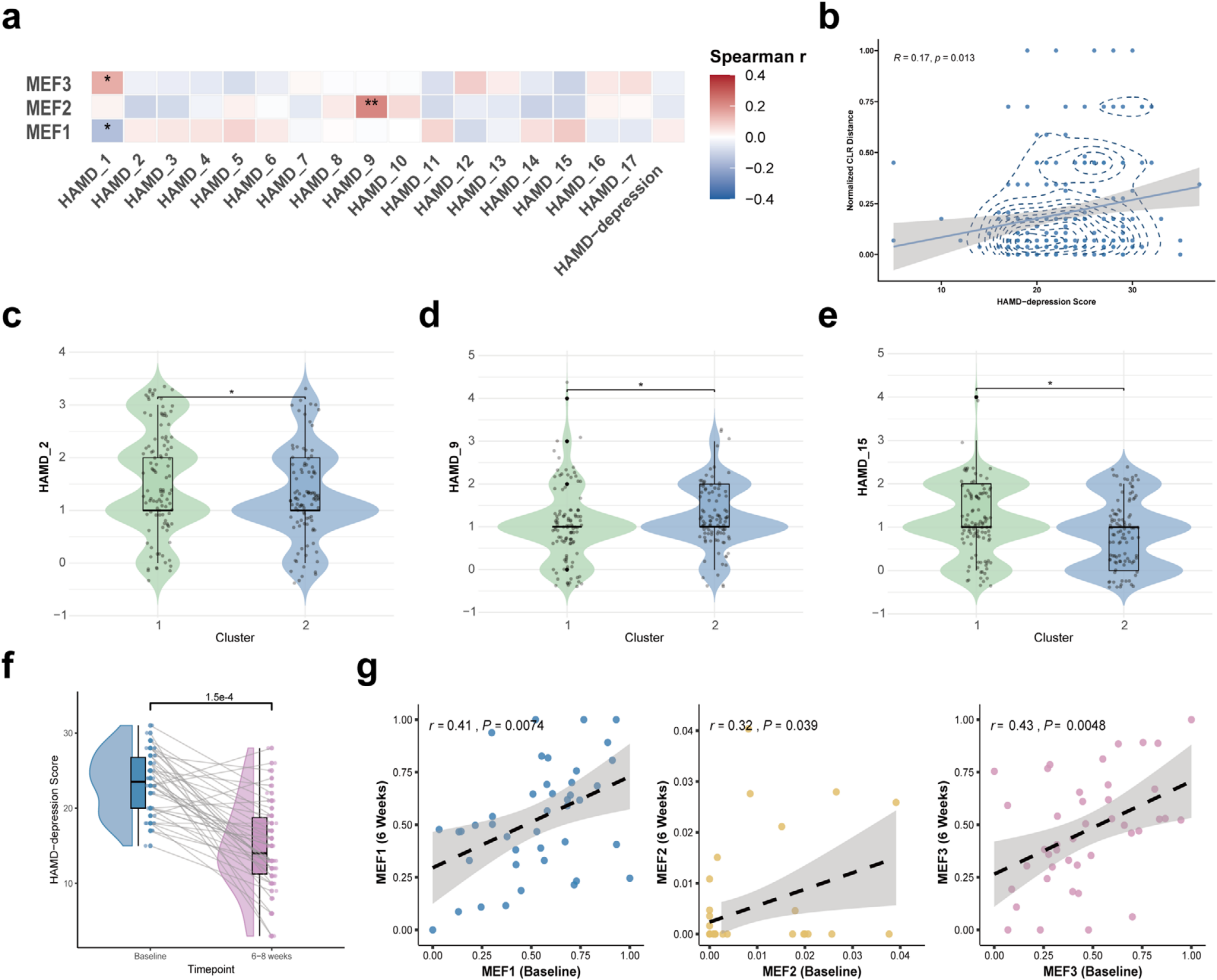
Symptom correlates and longitudinal dynamics of MEFs. (a) Heatmap shows Spearman correlations between MEF1‐3 scores and individual items on the 17‐item HAMD, including total depression severity (*n* = 230). (b) Scatter plot showing the correlation between the CLR‐normalized ecological deviation from the healthy baseline and HAMD total scores, indicating a significant positive association (*r* = 0.17, *p* = 0.013). (c–e) Violin–boxplots display differences in HAMD item scores (HAMD_2: guilt, HAMD_9: agitation, HAMD_15: hypochondriasis) across two MEF‐based clusters, with asterisks denoting significant group differences (Wilcoxon rank‐sum test, two‐sided; *n* = 230). (f) Paired boxplots show a significant reduction in HAMD‐depression scores from baseline to follow‐up among MDD participants. (g) Scatter plots show significant longitudinal correlations between baseline and follow‐up MEFs scores for MEF1 (*r* = 0.41, *p* = 0.0074), MEF2 (*r* = 0.32, *p* = 0.039), and MEF3 (*r *= 0.43, *p* = 0.0048), indicating intra‐individual temporal stability (paired Wilcoxon signed‐rank test, two‐sided; *n* =42).

Analysis of the associations between MEFs and clinical symptom severity revealed no significant relationships at the overall level (Figure S14a). However, certain MEFs were significantly associated with specific symptoms, including depressed mood (item 1) and agitation (item 9) of the Hamilton Depression Rating Scale (17‐item, HAMD‐17) (FDR *p* < 0.1; Figure 6a, Table S14). MEFs were also linked to peripheral biomarkers, including a positive correlation between MEF1 and prolactin, and a negative correlation between MEF2 and red blood cell count (FDR *p* < 0.05; Figure S14b). In light of the weak associations at the global symptom level, we next assessed individual‐level ecological divergence from health by computing Euclidean and orthogonal distances within the MEFs space. While Euclidean distance to the healthy reference profile showed no association with HAMD scores, orthogonal deviation from the healthy reference plane was positively correlated with symptom severity (*r* = 0.17, *p* = 0.013; Figure 6b), suggesting that greater divergence from health‐associated configurations reflects increased clinical burden.

Furthermore, to explore heterogeneity within MDD, k‐means clustering based on MEFs profiles identified two microbial subtypes (Figure S15), which differed significantly across multiple depressive symptoms. The most notable differences were observed in guilt, agitation, and hypochondriasis, suggesting potential subtype‐specific clinical relevance (Figure 6c–e). Finally, in a longitudinal analysis of 42 patients undergoing antidepressant treatment, a significant improvement in depressive symptoms was observed (Figure 6f), whereas MEFs expression remained largely stable (Figure S16). Nonetheless, moderate within‐subject consistency (*r* = 0.32–0.43, *p* < 0.05, Figure 6g) suggests short‐term pharmacological intervention does not substantially shift microbial ecological structure. Notably, baseline Wiredancer‐derived MEFs profiles failed to predict treatment response, which may be attributable to limited sample size and substantial clinical heterogeneity. Overall, Wiredancer still provides a robust and broadly applicable analytical tool for linking the ecological organization of the gut microbiome to clinical phenotypes in complex diseases.

### 2.9 Sensitivity and Robustness Analyses

Through a series of sensitivity analyses, we systematically assessed the robustness of our analytical framework across multiple dimensions, including preprocessing choices, model configurations, and cohort structures (Figures S17–S22). Applying a 0.01% abundance threshold had negligible effects on both within‐sample diversity and between‐sample similarity, as reflected by near‐identical Shannon diversity and highly overlapping cosine similarity distributions (Figure S17). Consistent patterns were observed across different prevalence thresholds (5%, 15%, and 20%), where both diversity and pairwise similarity structures remained highly concordant relative to the baseline (10%), with correlation coefficients exceeding 0.97 (Figure S18), indicating that the inferred ecological structure is largely insensitive to filtering stringency. Additionally, compared with conventional dimensionality reduction methods, MEF achieved higher explained variance (Figure S19), supporting its ability to more effectively capture the underlying ecological structure. To further evaluate model dependence on training strategy, we systematically compared three approaches: healthy‐only, all‐sample, and split‐training schemes. The healthy‐only strategy consistently demonstrated superior modeling performance and achieved higher discriminative capacity based on derived features relative to the other strategies. In addition, ICC analyses across different random initializations showed improved consistency and stability under this strategy, supporting its suitability as a reference modeling space (Figure S20). Analyses across cohorts and preprocessing strategies further revealed consistent model performance, minimal contribution from cohort identity, and high reproducibility under bootstrap resampling (Figure S21). In MEF space, disease samples consistently exhibited greater distances to group centroids compared to healthy controls across cohorts, whereas variability among healthy controls remained low and stable, providing a reliable baseline reference. This pattern persisted after covariate adjustment and was primarily associated with disease status rather than demographic or cohort‐related factors (Figure S22). Overall, these analyses support the robustness of the identified structure across analytical settings and cohort heterogeneity, suggesting that it captures a stable ecological signal rather than artifacts introduced by technical or study‐specific variation.

## 3 Discussion

To the best of our knowledge, this study utilizes the largest and most globally representative metagenomic dataset to date to establish and validate Wiredancer, a novel, scalable data‐driven framework that delineates the continuum of gut microbial states across health and disease. We discovered three stable and broadly applicable MEFs dominated by *Bacteroides uniformis*, *Prevotella copri*, and *Bifidobacterium adolescentis*, which capture the characteristic compositional patterns of the gut microbiota at the individual level. Moreover, MEFs also reflect inter‐individual differences in ecological stability, temporal variability, and dynamic fluctuations. Furthermore, validation in psychiatric disorder cohorts demonstrated that MEF variations were significantly associated with disease symptoms, cognitive function, and peripheral biomarkers, and also facilitated subtype stratification, underscoring the translational potential of Wiredancer in precision medicine.

Our research findings indicate that there are significant differences in gut microbiota composition between healthy and unhealthy individuals. In unhealthy populations, the balance of the gut microbiota may be disrupted due to a significant decrease in several important symbiotic species, including *Faecalibacterium prausnitzii* [40] and *Akkermansia muciniphila* [41]. These species are known to produce short‐chain fatty acids and exert anti‐inflammatory effects, indicating a link between systemic microbial imbalance and disease. In parallel, potentially pathogenic bacterial species such as *Klebsiella pneumoniae* [42] were significantly enriched, indicating that under conditions of dysbiosis, these bacterial communities may gain an ecological advantage and promote disease progression. It is worth noting that species‐level analysis revealed significant ecological differences that were difficult to identify at the genus level. For instance, although *Ruminococcus gnavus* [43] and *Ruminococcus lactaris* belong to the same genus, the former is enriched in unhealthy populations and has been found to be associated with various diseases, while the latter is mainly enriched in healthy populations. This opposite distribution trend emphasizes the necessity of using high‐resolution classification in microbiological research. Genus‐level aggregation may obscure clinically relevant microbiological characteristics, whereas species‐level analysis better captures heterogeneity within taxonomic units [44]. In addition, we found that microbial imbalance characteristics were highly correlated with the MEFs pattern, suggesting that gut microbiota disruption is not driven by changes in individual species, but rather reflects a systemic ecological restructuring [45].

Interestingly, the Wiredancer framework consistently extracted three MEFs across multiple repeated analyses, underscoring both the stability and reproducibility of this data‐driven approach. These three types of MEFs exhibit distinctly different compositional characteristics and ecological functional tendencies, supporting and expanding upon the classic enterotype theory [28]. Specifically, MEF1 is dominated by anaerobic symbiotic bacteria such as *Bacteroides uniformis* [46], resembling the traditional *Bacteroides*‐type gut microbiota; MEF2 is rich in fiber‐fermenting bacterial communities such as *Prevotella copri* [47] and *Eubacterium rectale*, exhibiting ecological characteristics similar to the *Prevotella*‐type; while MEF3 is represented by genera such as *Bifidobacterium adolescentis* [48] and *Collinsella aerofaciens*, reflecting characteristic intestinal ecological configurations. In contrast to previous genus‐level classification approaches [30, 49], Wiredancer identifies more discriminative microbial community structures at the species level, thereby enhancing the ability to characterize individual differences. Notably, Wiredancer revealed that MEF1 and MEF2 form a dynamic equilibrium in healthy individuals, a balance that is disrupted in disease conditions, with MEF1 being upregulated and MEF2 downregulated. Interestingly, MEF3 can flexibly shift toward either MEF1 or MEF2, suggesting that it represents an intermediate ecological configuration between these two dominant patterns. This finding reinforces the central concept of Wiredancer, namely that ecological balance and constraint jointly define community homeostasis. Unlike previous “core functional groups” that viewed healthy and disease‐associated communities as opposing binary structures [39], our results indicate that host health status is not simply determined by the competitive balance between disease‐associated and healthy‐associated microbial communities, but rather stems from a community steady state shaped by multiple ecological expressions. In other words, health is not the dominant state of a particular type of microbiota, but rather a multidimensional, multi‐module synergistic microecological balance [50]. Our findings are consistent with previous studies, in which an overabundance of *Bacteroides* has been identified as a key disease‐associated feature [51]. The *Bacteroides*‐dominated MEF1 is significantly upregulated and exhibits increased variability under disease conditions, reflecting a “resilient yet impaired” core homeostasis deviation [33]. In contrast, MEF2 shows higher vulnerability as a core ecological factor, marked by niche displacement and a tendency toward functional collapse. This phenomenon further supports the view that there is strong “mutually exclusive” ecological competition between *Prevotella*‐type and *Bacteroides*‐type communities [33, 52], highlighting the importance of species‐level resolution in capturing such ecological dynamics.

We further validated that MEFs derived from Wiredancer exhibit high stability in age‐related changes [53, 54], inter‐individual variability, and ecological transition patterns between healthy and unhealthy states. MEF1 was higher in unhealthy individuals across all age groups compared to the healthy group, while MEF2 remained consistently downregulated. This antagonistic expression pattern remained stable across different age groups, further validating the long‐term disruption of microbial homeostasis in disease states and demonstrating a high degree of consistency between disease and microbial dysbiosis. Interestingly, in healthy populations, a distinct expression inflection point was observed around the age of 60–70, which coincides with the retirement age in many countries [55]. This phenomenon may reflect the potential impact of lifestyle changes on gut ecological status, warranting further systematic evaluation in future studies [56]. Additionally, we observed a significant increase in interindividual variability of disease‐related factors compared to health‐related factors, consistent with previous studies reporting enhanced microbial community volatility under disease conditions [57]. It is worth emphasizing that individual MEF expression exhibits high stability over time, demonstrating potential as a marker of individual microbiota status [58]. Although this expression state undergoes slight drift over time, its changes exhibit a degree of predictability, offering a potential pathway for developing personalized intervention strategies based on microbial status.

Furthermore, the generalizability of Wiredancer was systematically validated in one of the largest available cohorts of individuals with severe major psychiatric disorders, including MDD, SZ, and BD. All three disorders exhibited consistent and significantly shifted MEFs expression patterns relative to the healthy baseline, with substantial overlap in microbiota dysbiosis features across diagnoses. These findings further support the conceptualization of psychiatric disorders as points along an ecological continuum [59, 60], as opposed to the conventional paradigm that treats them as mutually exclusive diagnostic categories. While previous studies based on discrete clinical variables have identified shared features across psychiatric disorders [12], our findings suggest that these similarities may reflect common functional expression patterns within the gut microbiota. We also observed disease‐specific differences in the variability of MEFs expression, indicating that microbiota disruption not only manifests as shifts in average levels but also involves alterations in the dynamic structure within communities [61]. This variability may reflect the dynamic instability of psychiatric disorders during their pathological progression, providing potential clues for understanding their biological heterogeneity and subtype evolution. In addition, investigating the extent of MEFs disruption in relation to disease course provides important insights into clinical progression and therapeutic intervention [62]. Compared to first‐episode, untreated individuals, non‐first‐episode MDD and SZ patients exhibited significantly enhanced variability in MEF1, reflecting an exacerbation of microbial imbalance as the disease progresses. Notably, MEF2 exhibited divergent trajectories across the two disorders: its expression progressively increased with disease duration in MDD, whereas it showed a continuous decline in SZ. Given that non‐first‐episode patients typically receive long‐term pharmacological treatment and that treatment mechanisms vary across different psychiatric disorders [63], this change may reflect differing effects of disease intervention pathways on the microbiota, suggesting potential underlying mechanisms of microbiota–host interactions in disease evolution. Specifically, MDD exhibits fluctuating MEF2 expression that increases with disease progression, while SZ shows an early divergent, late convergent microbiota succession trajectory. These dynamic trajectories, revealed through the Wiredancer framework, highlight its ability to resolve disease‐specific ecological pathways that conventional static models may overlook.

Finally, we assessed the clinical relevance of MEFs and found that they showed stable associations with symptom dimensions, peripheral blood biomarkers, and deviations from healthy baselines, systematically revealing these relationships for the first time from a systemic microbiological perspective. Consistent with previous studies, the MEFs model derived from NMF can also be used to identify clinically significant subtypes [64], demonstrating its potential for achieving refined classification in populations with mental disorders. However, compared with recent brain imaging‐based factor models [32], we have not yet achieved effective prediction of treatment response. This limitation may reflect the high heterogeneity of MDD, suggesting the need to integrate multi‐omics information to enhance predictive capabilities. Additionally, sample size limitations may have impacted our ability to identify the dynamic diversity of microbial factors [6]. Future research should focus on overcoming these challenges to advance the translational application of personalized diagnostic and intervention strategies based on microbiota characteristics.

This study has certain advantages in terms of sample size, model construction, and multi‐disease validation, but it still has several limitations. First, the analyses primarily relied on bacterial metagenomic data and should be extended to include multi‐kingdom microbiota and multi‐omics layers to more comprehensively capture gut ecosystem complexity [65, 66, 67, 68, 69, 70]. Second, longitudinal data across the health‐disease continuum remain limited. Integrating follow‐up data from multiple disease cohorts may help to delineate the dynamic trajectories of community homeostasis during disease progression. Meanwhile, the causal effects and underlying biological mechanisms of the inferred ecological factors still require further confirmation through animal experiments and other functional validation strategies [71]. Finally, although the MEFs framework demonstrated robust cross‐disease generalizability, its practical clinical utility in predicting treatment responses still requires further validation and optimization in larger prospective longitudinal cohorts.

## 4 Conclusion

In this study, we developed Wiredancer, a scalable modeling framework that consistently extracts three robust MEFs, thereby mapping the health‐disease continuum of the global gut microbiome. Systematic validation in large psychiatric disorder cohorts demonstrated that the framework can closely link microbial ecological patterns with clinical phenotypes. Wiredancer translates microbial heterogeneity into continuous ecological dimensions, offering a generalizable paradigm for precision medicine and a powerful avenue for advancing microbiome‐based diagnostics and interventions.

## 5 Experimental Section

### 5.1 Data Collection

We integrated a global fecal metagenomic sequencing resource spanning 36 countries and comprising a total of 20,178 samples, covering healthy and various unhealthy states. The public dataset is sourced from curated Metagenomic Data [72] and large population studies conducted in regions such as Canada, the United States, and China [19, 73, 74, 75, 76]. Only cohorts with a complete sequencing platform and sample collection metadata, passing quality control were included. To focus on major psychiatric disorders, we curated existing metagenomic datasets with clearly annotated diagnostic labels for SZ, MDD, and BD, removing duplicate entries and records lacking key information. In parallel, as part of the Brain–Gut Health Initiative (BIGHI) [77], we established a local clinical sub‐cohort comprising 609 participants, including 36 with chronic SZ, 263 with MDD, 90 with adolescent‐onset BD, and 220 matched healthy controls. The standardized data collection includes demographic information, anthropometric measurements, blood biochemistry and inflammatory markers, cognitive assessments, and disease‐specific severity scales [77]. Psychiatric diagnoses were confirmed by senior psychiatrists using the Structured Clinical Interview based on DSM‐IV‐TR or DSM‐5 criteria, depending on the time and site of assessment. All participants signed written informed consent forms, and the study was approved by the institutional ethics committee (Approval No. (2025) 056) and conducted in accordance with the Declaration of Helsinki [78]. All metagenomic data were preprocessed and annotated using standardized workflows implemented in the BioBakery suite, including sequence quality control, taxonomic profiling, functional profiling, and batch effect correction [79]. This integrated dataset provides the foundation for subsequent modeling of MEFs, assessment of cross‐cohort generalizability, and analysis of longitudinal stability. Detailed sample composition, quality control criteria, and analytical procedures are provided in Supplementary Materials.

### 5.2 Stool and Phenotypic Data Collection and Processing

Fecal and peripheral blood samples were collected on the day of clinical assessment according to a standardized operating procedure. Participants fasted the night before and had their samples collected under sterile conditions the following morning. Fecal samples were immediately frozen at −80°C and subsequently used for microbial DNA extraction and metagenomic sequencing. Peripheral blood samples were used to measure routine biochemical and inflammatory markers, including 35 blood biochemical indicators such as C‐reactive protein, homocysteine, and superoxide dismutase [11]. Additionally, demographic information (age, sex, years of education), anthropometric data (height, weight, and BMI), and structured clinical assessment information were recorded. Detailed procedures for sample handling and sequencing are provided in Supplementary Materials.

### 5.3 Enterotype Inference and Community Clustering

Enterotype‐like structures were identified using PAM and DMM, two complementary clustering methods that enabled detection of compositional patterns [28, 29]. Cluster numbers were evaluated using silhouette width and Calinski–Harabasz statistics, and robustness was assessed by repeated subsampling at varying random fractions. To mitigate disease‐related global bias effects and highlight the specific patterns of microbial variation in healthy individuals, we employed a sparse‐constrained contrastive principal component analysis method, setting healthy samples as the foreground and aggregated disease samples as the background [80]. The resulting contrastive latent space highlighted representative or relatively stable variance components in the healthy gut microbiota. To further characterize the discrete patterns in this space, we applied K‐means clustering to the contrast components and determined the optimal number of clusters using contour coefficient analysis [49]. Full methodological details and parameter settings are provided in Supplementary Materials.

### 5.4 Wiredancer Modeling Framework

Within the Wiredancer framework, MEFs were extracted using similarity‐constrained NMF [81], which aims to decompose the input matrix *X* into the product of two non‐negative matrices *W* and *H*, such that:

X≈WH

Because the NMF framework requires all elements of the input matrix to be non‐negative, X was defined as a normalized species‐level relative abundance matrix. This non‐negative matrix was then decomposed into two lower‐dimensional matrices: W, a sample‐by‐factor matrix representing the factor profile of each sample, and H, a factor‐by‐taxon matrix representing the taxonomic composition of each microbial ecological factor. The number of factors reflects the structure of the microbial community. The parameter *k* represents the number of latent factors. To enhance interpretability and structural preservation, a sparsity constraint [64] and a graph regularization term [82] were further introduced. The model was used to obtain a low‐dimensional latent representation of the gut microbiota in healthy individuals. To determine the optimal number of latent factors and regularization parameters, a two‐stage model selection strategy was adopted [33]. In the first stage, we evaluated multiple candidate factor numbers (*k* ∈  [2, 10]) under a non‐regularized setting. For each value of *k*, fivefold cross‐validation was performed and repeated 100 times to reduce the influence of random data partitioning. Evaluation metrics included clustering stability assessed by the cophenetic correlation coefficient. The factor number yielding the highest stability without further improvement in model performance was selected as the optimal rank. In the second stage, regularization terms were introduced based on the selected optimal rank, and joint tuning of the sparsity coefficient (*α*) and graph regularization coefficient (*λ*) was performed. An initial coarse grid search was conducted to identify a high‐performing parameter region, with the sparse regularization coefficient *α* ∈ {0, 0.001, 0.01, 0.05, 0.1, 0.2, 0.5, 1} and the graph regularization coefficient *λ* ∈ {0, 0.01, 0.1, 1, 5, 10, 15, 20}. Within the top 10% parameter combinations identified from the grid search, Bayesian optimization was further applied for fine‐scale parameter search [83]. During optimization, the cross‐validated mean explained variance was used as the objective function. A Gaussian process regression model was used to model the response surface, with the Upper Confidence Bound acquisition function guiding sampling to identify an optimal parameter balancing sparsity, structural fidelity, and reconstruction accuracy.

### 5.5 MEFS Analysis

Within the Wiredancer framework, the model is trained on an external healthy control dataset to learn a fixed set of ecological factors defined over microbial species, providing a stable reference due to the lower variability of the healthy microbiome. External disease datasets were projected into this space, while independent psychiatric cohorts and the BIGHI cohort, including patients, healthy controls, and longitudinal follow‐up samples, were used to validate its stability and generalizability. Factor representations for each sample were estimated via non‐negative least squares, enabling unified representation in the shared latent space [33]. We normalized the W and H matrices obtained from the NMF decomposition to characterize the overall factor composition of each sample and the relative contribution of each factor, respectively. Additionally, we extracted individual‐level factor expression profiles from the normalized matrices to compare differences between healthy and diseased populations. Both ecological composition diversity and factor discriminative ability were evaluated based on the normalized W matrix derived from NMF decomposition, where Shannon entropy quantified ecological composition diversity and logistic regression assessed factor discriminative ability. In addition, ecological factors were also linked to functional potential by calculating MEF‐specific metabolic pathway scores derived from HUMAnN outputs, enabling functional interpretation of factor‐level alterations. To explore the associations between ecological factors and microbial taxonomic shifts, species‐level t‐statistics reflecting group differences were computed, and Pearson correlations were performed between these statistics and the taxon loadings across factors [84]. Further methodological details and supporting analyses are described in Supplementary Materials.

### 5.6 Stability Analysis of MEFs Profiles

To examine the expression patterns and variability of MEFs derived from Wiredancer across populations, we first stratified healthy individuals into age groups and calculated the mean MEF expression within each group to assess age‐associated ecological trends. To quantify inter‐individual variability, we calculated the lnCVR for each MEF between disease and healthy groups. This metric incorporates both variance and mean differences between groups, where *s*_p_ and *s*_c_ are the sample standard deviations, *m*_p_ and *m*_c_ are the sample means, and *n*_p_ and *n*_c_ are the sample sizes of the patient and control groups, respectively [62].

$$\ln CV\;R=\ln\left(\frac{\frac{s_{p}}{m_{p}}}{\frac{s_{c}}{m_{c}}}\right)+\frac{1}{2(n_{p\;}-\;1)}-\frac{1}{2(n_{c}-\;1)}$$

We also calculated the cosine similarity between individuals at different time points based on normalized MEFs expression profiles, compared differences in temporal stability among groups, and analyzed the relationship between similarity and follow‐up time intervals using a linear regression model [85]. Additionally, we constructed a discrete‐time Markov chain model by assigning each sample to an MEF‐dominant state and derived group‐specific empirical transition matrices from repeated measurement data to characterize ecological state transitions and reshaping patterns across different groups [86]. To further assess the predictability of MEF dynamics, we employed SVR models [32]. For individuals with at least three time points, final MEF expression was predicted from preceding observations using subject ID and time as predictors. Model performance was evaluated by Spearman correlations between predicted and observed values. Additional methodological details and supporting analyses are provided in Supplementary Materials.

### 5.7 MEFs Profiling in Major Psychiatric Disorders

We profiled MEFs expression levels in patients with MDD, SZ, and BD compared to healthy controls. Differences in MEFs scores between groups were compared using the Wilcoxon rank‐sum test. To visualize ecological differences between diagnostic groups, we projected individual samples into a three‐dimensional MEFs space and calculated the centroids of each group to demonstrate changes in microbial community structure among the four populations [32]. To quantify intra‐group compositional variability, we calculated the lnCVR for each MEF, as described previously. lnCVR values were computed separately for the BD, MDD, and SZ groups relative to the healthy group [62]. Within each disorder, we further compared variability between non‐first‐episode and first‐episode subgroups to examine the potential impact of disease duration and progression on ecological stability. In addition, cognitive function was measured across multiple dimensions using the MATRICS Consensus Cognitive Battery [87]. Cognitive deficits in MDD and SZ patients were quantified using Cohen's d effect size calculations compared to healthy controls. To analyze the relationship between gut microbiota composition and cognitive performance, we performed CCA between individual‐level MEFs loadings and scores on each cognitive dimension [88]. Additionally, Pearson correlation coefficients were calculated between each MEF and cognitive dimension to assess the strength and direction of their association.

### 5.8 MEFs and Clinical Correlates

We employed three complementary strategies to analyze the association between MEFs and the HAMD‐17 total scores and item‐level scores [89]. First, we calculated the Spearman correlation coefficient between each MEF and each HAMD item to identify symptom‐level correlations. Second, in the original factor space, we calculated the Euclidean distance between each MDD patient and the average MEFs expression profile of the healthy group and performed a correlation analysis between this distance and HAMD scores. Third, because the normalized MEFs were compositional and subject to the constant‐sum constraint in joint analysis of the three factors, we applied the centered log‐ratio (CLR) transformation in the Wiredancer latent space to enable appropriate geometric analysis in Euclidean space. In the transformed space, we constructed a geometric reference plane based on the healthy control samples and further calculated the shortest vertical distance from each MDD patient to the reference plane, correlating it with HAMD scores as an interpretable metric for deviation from the healthy ecological baseline [90]. To investigate Wiredancer‐derived heterogeneity within MDD, we performed unsupervised clustering on CLR‐transformed MEFs values using K‐means, with the optimal number of clusters selected by maximizing silhouette width. Clinical variables, including HAMD subscales, were compared across identified clusters using Wilcoxon rank‐sum tests. To further characterize microbial subtypes, differential abundance analyses were performed to identify distinct taxonomic, gene, and functional signatures between subgroups. Moreover, to assess the potential of MEFs in predicting treatment response, we established a predictive model based on follow‐up data collected at 6 to 8 weeks from patients with MDD. Using the MEFs expression profile of the healthy group as a reference, we quantified the degree of deviation in patients and combined it with MEF‐derived features to perform modeling using partial least squares regression. Model performance was evaluated using leave‐one‐out cross‐validation, and the predictive results were correlated with actual symptom improvement [32].

### 5.9 Bioinformatics Analysis

Microbial community analysis was performed using a standard workflow. The microbial relative abundance data were adjusted using the MMUPHin [91] software package to control for potential confounding factors between cohorts. Alpha diversity was assessed using the Shannon index and Simpson index to measure species richness and evenness within samples [92]. Beta diversity was calculated based on Bray–Curtis distances and visualized using principal coordinate analysis to characterize differences in community composition between samples. The statistical significance of intergroup Beta diversity differences was tested using multivariate analysis of variance (PERMANOVA) with 9999 permutations [10]. Differential abundance analysis of microbial taxonomic units was performed using multivariate linear modeling in MaAsLin2 to identify microbial communities significantly associated with group status [93]. Extended methodological information is available in Supplementary Materials.

### 5.10 Statistical Analysis

All statistical analyses were performed using R (version 4.3.2). Species‐level relative abundance data were normalized, and the CLR transformation was applied where appropriate for compositional analyses. Categorical variables were compared using chi‐square tests. For continuous variables, two‐sided *t*‐tests were used when assumptions of normality and homogeneity of variance were met; otherwise, Wilcoxon rank‐sum tests were applied. Data are presented as mean ± SD or median, as appropriate. Correlations were assessed using Pearson or Spearman methods, depending on data distribution. Sample sizes varied across analyses and are indicated where appropriate. *p*‐values were adjusted using the Benjamini–Hochberg method to control for multiple comparisons. A two‐sided *p* < 0.05 was considered statistically significant unless otherwise specified.

## Author Contributions

BZ conceptualized the study, designed the methodology, conducted formal analysis, and drafted the original manuscript. YH, SC, WW, and LL developed the methods, curated data, and contributed to manuscript review and editing. YD, XDL, RH, MG, ZL, and SW assisted with data curation and manuscript revisions. HL and CL managed the collection of clinical data. JZ, DX, XBL, and YN supported methodological development, data processing, and study supervision. XS, FW, and KW provided overall supervision, secured funding, and reviewed and edited the final draft. All authors read and approved the manuscript.

## Funding

This work was supported by the National Key Research and Development Program of China (2023YFC2414500, 2023YFC2414504, 2025YFC3410000, and 2025YFC3410005), the National Natural Science Foundation of China (82271953 and 82301688), the Key Research and Development Program of Guangdong (2023B0303020001 and 2023B0303010003), the Natural Science Foundation of Guangdong Province (2024A1515013058, 2025A1515010507, and 2023A1515011383), the Guangdong Key Laboratory of Battery Safety at Guangzhou Institute of Energy Testing (2019B121203008‐KJ‐2024‐040/KJ‐2024‐041), the Science and Technology Program of Guangzhou (2025A03J3357), the Clinical Collaboration Project on Integrated Traditional Chinese and Western Medicine for Major and Difficult Diseases (Bipolar Disorder, ZDYN‐2024‐A‐121), the Research Capacity Improvement Project of Guangzhou Medical University (2024SRP200), Guangzhou Key Clinical Specialty (Clinical Medical Research Institute), the Announcement and Leading Science and Technical Foundation of Guangzhou Civil Affairs (GCAAL2022001), and the Guangzhou Planned Project of Science and Technology (2023B04J0106 and 2025B04J0011).

## Ethics Statement

The study protocol was approved by the Ethics Committee of the Affiliated Brain Hospital of Guangzhou Medical University (Approval No. (2025) 056), and written informed consent was obtained from each subject or their legal guardian before the study. This study was conducted in accordance with the Declaration of Helsinki.

## Conflicts of Interest

The authors declare no conflicts of interest.

## Supporting information




**Supporting File**: advs75670‐sup‐0001‐SuppMat.docx.

## Data Availability

Datasets from the curatedMetagenomicData repository are available at: https://github.com/waldronlab/curatedMetagenomicData. Additional cohorts used in this study can be accessed through publicly available databases. Clinical cohort samples collected by our laboratory are not publicly available due to privacy considerations but can be accessed upon reasonable request in de‐identified form. Further information is available from the corresponding author upon request. Source data for all main figures are provided as supporting data files. Source data are provided with this paper. Source codes used in this study are available from GitHub (https://github.com/zbyloveyj/SCUT_Minilab_MEF).
