# Supplementary material for: Dissecting the Ecological Structure of Health and Disease in the Global Gut Microbiome
Source: Adv Sci (Weinh). 2026 May 19;13(44):e17087. doi: 10.1002/advs.202517087 (PMC13335992; doi:10.1002/advs.202517087)
Supplement: Supplementary file 1 [file ADVS-13-e17087-s001.docx]

**Supporting Information**

**Wiredancer Maps the Health–Disease Continuum in Global Gut Microbiome Landscape**

Baoyuan Zhu, Shuhao Chen, Yunheng Diao, Wei Wang, Yuanyuan Huang, Liqin Liang, Xiaodan Lu, Rui Han, Minxin Guo, Zhaobo Li, Shuhong Wang, Hehua Li, Chenyu Liu, Jing Zhou, Dongsheng Xiong, Xiaobo Li, Yuping Ning, Xuetao Shi*, Fengchun Wu*, Kai Wu*

**Supplemental Methods**

**1.1 Participant recruitment**

This study included four groups of participants: individuals with schizophrenia, adolescents with bipolar disorder experiencing a depressive episode, patients with first-episode and drug-naïve major depressive disorder, and healthy controls. All participants were recruited from the Guangzhou Brain Hospital affiliated with Guangzhou Medical University and surrounding communities. The study was approved by the institutional ethics committee and conducted in strict accordance with the principles of the Declaration of Helsinki. Written informed consent was obtained from all participants or their legal guardians after a full explanation of the study objectives, procedures, and potential risks.

All participants were required to meet the following general inclusion criteria: Han Chinese ethnicity, right-handedness, normal or corrected-to-normal vision, no major physical illnesses, neurological disorders, or cognitive impairment, and an intelligence quotient of no less than 80 as assessed by standardized intelligence scales. Participants were required to understand the study procedures and comply with clinical assessments and biological sampling. General exclusion criteria included: diagnosis of other major psychiatric disorders according to the Diagnostic and Statistical Manual of Mental Disorders (DSM) IV-TR (e.g., schizoaffective disorder, pervasive developmental disorders, or neurodegenerative diseases); history of organic brain diseases (e.g., encephalitis, epilepsy, stroke), head trauma, or systemic illness affecting cognition; pregnancy or breastfeeding; use of antibiotics, probiotics, gut microbiota-directed therapies, laxatives, antidiarrheals, or other gastrointestinal interventions within three months; recent respiratory, urinary, or gastrointestinal infections within the past month; history of substance dependence or abuse (excluding nicotine); high suicide risk or recent electroconvulsive therapy; a body mass index (BMI) over 28; extreme dietary patterns (e.g., strict vegetarians or severe picky eaters); contraindications for magnetic resonance imaging; or any other medical condition deemed inappropriate for study participation by the investigators.

Participants in the schizophrenia group were inpatients aged 18-65 years whose treatment regimen had remained unchanged for at least two weeks, indicating clinical stability. Diagnosis was made by two licensed psychiatrists based on the DSM-IV-TR, confirmed using a structured clinical interview. Symptom severity was evaluated using the Positive and Negative Syndrome Scale (PANSS), with a total score of no less than 60 and at least two positive symptom items rated at or above moderate severity. To ensure stability, the percentage change in total scale score within the two weeks prior to enrollment was required to be less than 20%. Individuals with comorbid psychiatric disorders, severe somatic illness, gastrointestinal disease, or history of microbiota intervention were excluded.

The adolescent bipolar disorder group included individuals aged 12-18 years who met the diagnostic criteria for bipolar disorder in the depressive episode as defined by the DSM-5. Diagnosis was confirmed by trained psychiatrists using a structured clinical interview. Participants were required to be in a depressive episode at the time of enrollment, with no current or recent manic episodes, mixed states, or rapid cycling. Exclusion criteria included any comorbid psychiatric diagnosis, recent gastrointestinal infections or surgery, organic brain diseases, antidepressant treatment within the past three months, use of antibiotics or probiotics within the past month, history of substance misuse, previous modified electroconvulsive therapy, suicide risk, or inability to cooperate with study procedures.

Patients with major depressive disorder (MDD) were aged 18-45 years and met the DSM-5 diagnostic criteria, confirmed by structured clinical interviews conducted by trained psychiatrists. Both first-episode, drug-naïve and previously treated individuals were included. Participants were required to have a Hamilton Depression Rating Scale (17-item) score of at least 17 and a Young Mania Rating Scale score of no more than 5. Individuals with current or past manic symptoms, psychotic features, or comorbid psychiatric disorders were excluded.

Healthy control participants were recruited from Guangzhou and neighboring communities through posters, online platforms, and hospital referrals. Controls were matched with patient groups on age, sex, education level, and intelligence. Exclusion criteria included: personal history of psychiatric illness, family history of psychiatric disorders among first-degree relatives, and any current or past psychiatric diagnosis confirmed through a structured clinical interview. Individuals were also excluded if they had used any psychotropic or gut microbiota–related interventions in the past three months, experienced recent infections, or had chronic systemic diseases. Participants were required to have a BMI below 28 and regular dietary habits, with no extreme dietary restrictions or behaviors.

All participants underwent standardized clinical interviews, neuropsychological assessments, physical examinations, and biospecimen collection. Psychiatric symptoms were evaluated using the PANSS for schizophrenia, the 17-item Hamilton Depression Rating Scale for depressive symptoms, and the Young Mania Rating Scale for manic symptoms. Cognitive function was assessed using the MATRICS Consensus Cognitive Battery (MCCB), which covers five key cognitive domains: processing speed, attention and vigilance, working memory, verbal learning, and visual learning. All cognitive assessments were conducted individually in a quiet testing environment by trained research assistants.

Biological samples included same-day fecal samples for metagenomic sequencing and peripheral blood samples for biochemical and inflammatory marker analyses. All procedures for sample collection and processing adhered to rigorous quality control protocols to ensure scientific validity and data comparability across study groups.

**2.1 Fecal collection and DNA extraction and processing**

Stool and peripheral blood samples were collected on the day of clinical symptom assessment, and participants were asked to fast for at least 12 hours before sampling, which was done by a professional researcher under aseptic conditions from 7:00 a.m. to 9:00 a.m. the following morning. Fecal samples were frozen at -80°C immediately after collection for subsequent microbial DNA extraction, which was achieved by chemical lysis combined with mechanical fragmentation, followed by phenol-chloroform extraction to remove proteins, RNase treatment to degrade RNA, and ethanol precipitation to obtain purified genomic DNA. Samples were tested for concentration and integrity. DNA samples that passed the concentration and integrity tests were randomly interrupted into approximately 350 bp fragments using a Covaris sonicator to enter the library construction process. Library construction included end repair (T4 DNA polymerase and polynucleotide kinase), addition of A-tail at the 3' end, Illumina junction ligation, and double-end size screening using Agencourt SPRI magnetic beads to remove fragments that did not meet the length requirement. Qualified libraries were amplified by PCR and quantified using a Qubit 3.0 fluorometer, and insert fragment size was determined by an Agilent 5400 system. The libraries were immobilized on FlowCell chips by bridge PCR amplification, and finally sequenced by double-end 150 bp (PE150) high-throughput sequencing on the Illumina NovaSeq platform, generating raw data in FASTQ format, which contains sequence information and quality assessment indexes.

**2.2 Blood sample collection and processing**

Serum biochemical markers were assessed using standardized clinical procedures to evaluate inflammatory status, oxidative stress, and metabolic health. Serum C-reactive protein (CRP) levels were measured using an immunoturbidimetric assay. In this method, CRP in the sample forms antigen–antibody complexes with anti-CRP serum, and the intensity of light scattered at 340 nm is directly proportional to the CRP concentration. Fresh, non-hemolyzed serum samples were used for analysis. All reagents were obtained from Beijing Leadman Biochemical Co., Ltd. and stored at 2-8°C. Serum homocysteine (Hcy) levels were determined enzymatically using the SMT (S-adenosylhomocysteine hydrolase-mediated transformation) method. Following the release of free Hcy, adenosine is generated and hydrolyzed into hypoxanthine and ammonia. The rate of NADH consumption, catalyzed by glutamate dehydrogenase, is proportional to Hcy concentration. Both fresh non-hemolyzed serum and heparin-anticoagulated plasma samples were acceptable. Reagents were provided by Beijing Strong Biotechnologies, Inc., and stored at 2–8°C protected from light. Superoxide dismutase (SOD) activity was assessed using the pyrogallol autoxidation method, which quantifies the inhibition of pyrogallol autoxidation in alkaline conditions by SOD-mediated conversion of superoxide radicals (O2−•) to hydrogen peroxide and oxygen. The degree of inhibition is inversely proportional to SOD activity. Fresh, non-hemolyzed, non-lipemic serum or plasma samples were used. Kits were supplied by Fujian Fuyuan Biotechnology Co., Ltd., and stored at 2–8°C protected from light.

In addition, comprehensive metabolic and clinical chemistry profiles were evaluated. Complete blood count measurements included white blood cells (WBC), neutrophils (NEU), lymphocytes (LYM), monocytes (MON), eosinophils (EOS), basophils (BAS), red blood cells (RBC), hemoglobin (HGB), and platelets (PLT). Liver function tests comprised gamma-glutamyl transferase (GGT), alanine aminotransferase (ALT), aspartate aminotransferase (AST), alkaline phosphatase (ALP), total protein (TP), albumin (ALB), and globulin (GLO). Renal function markers included uric acid (UA), blood urea nitrogen (BUN), and creatinine (CRE). Lipid metabolism was assessed using triglycerides (TG), total cholesterol (TC), high-density lipoprotein cholesterol (HDL), and low-density lipoprotein cholesterol (LDL). Glucose homeostasis was evaluated based on fasting glucose (GLU), fasting insulin (INS), C-peptide (CPEP), and glycated hemoglobin (HbA1c). Hormonal profiles included thyroid-stimulating hormone (TSH), free triiodothyronine (FT3), free thyroxine (FT4), estradiol (E2), and prolactin (PRL). All measurements were conducted following standardized clinical biochemistry and hematology protocols in certified hospital laboratories, with rigorous internal quality control to ensure data accuracy and consistency.

**2.3 Metagenomic analysis**

All raw metagenomic sequencing data were uniformly preprocessed and taxonomically profiled using the established BioBakery pipeline developed by the Huttenhower Lab at Harvard University. These standardized preprocessing steps generated the input abundance matrices for downstream modeling within the Wiredancer framework. Initially, Trimmomatic was applied to trim bases with quality scores below Q20 and remove sequencing adapters, discarding reads shorter than 50 nucleotides. High-quality reads were then aligned to the human reference genome (GRCh38) using Bowtie2 to eliminate host-derived sequences. Taxonomic profiling was performed at the species level using MetaPhlAn3 (v3.0) based on clade-specific marker genes, with low-abundance noise (< 0.01%) smoothed. To reduce sparsity and enhance statistical robustness, only taxa present in more than 10% of samples were retained for downstream analysis. Batch effects across cohorts or sequencing runs were corrected using the MMUPHin method. The entire workflow was executed with consistent software versions and parameters via multi-threaded automated scripts, accompanied by detailed logs and quality control reports to ensure analytical reproducibility and robustness.

**3.1 Classical enterotype analysis**

To systematically characterize the macroscopic compositional structure of the gut microbiota in healthy individuals and to evaluate its consistency with traditional “enterotype” concepts, two commonly used clustering algorithms were applied to the standardized relative abundance matrix restricted to healthy samples: (1) Partitioning Around Medoids (PAM), using the Jensen-Shannon divergence to quantify compositional dissimilarity between samples; (2) Dirichlet Multinomial Mixture (DMM) models, implemented via the DirichletMultinomial package in R, aimed at capturing potential discrete compositional states. For each method, the number of clusters (k) was iteratively optimized within a pre-specified range: PAM clustering was evaluated using the Calinski-Harabasz index and average silhouette width, while DMM models were assessed using the Laplace approximation, Bayesian Information Criterion (BIC), and Akaike Information Criterion (AIC).

To validate the robustness of clustering results, subsampling was performed at 5%, 25%, 50%, 75%, and 100% of the original dataset, and the clustering procedures were repeated accordingly. Clustering consistency was evaluated by calculating metrics such as the adjusted Rand index and normalized mutual information across replicates, and the reproducibility of the optimal number of clusters was tracked across sampling proportions to distinguish true structural signals from sample size sensitivity.

**3.2 Contrastive Learning-Based Community Clustering**

Given that conventional enterotype clustering may be confounded by global shifts commonly present in disease cohorts (e.g., reductions in diversity or functional deviation), which can obscure finer-scale patterns of coordinated variation within the healthy population, a sparsity-constrained contrastive principal component analysis (cPCA) was further introduced. In this approach, healthy individuals were defined as the foreground (target) and combined disease samples were used as the background. The method seeks to maximize variance in the foreground while suppressing variance in the background in the projected low-dimensional space, thereby extracting compositional axes that are more pronounced or stable in healthy individuals. The sparsity parameter was optimized via grid search to balance target-specific variance explanation and feature interpretability. The resulting foreground contrastive components served as initialization directions for subsequent modeling of Microbial Ecological Factors. To further group individuals based on these components, K-means clustering was performed in the contrastive space, with the optimal number of clusters determined by average silhouette width.

**4.1 Evaluation of the discriminative power of MEF**

We first performed univariate logistic regression analyses for each factor, with disease status coded as a binary outcome (healthy = 0, disease = 1) and factor expression values derived from the row-normalized sample-level factor matrix as predictors. Because the three factors are compositional and sum to one, they are linearly dependent. To reduce multicollinearity while preserving interpretability, we ranked individual factors based on their discriminative performance, assessed using 5-fold cross-validated area under the curve (AUC), together with the statistical significance of between-group differences, and retained the two most informative factors for downstream modeling. These two selected factors were then entered into a multivariable logistic regression model to evaluate their joint ability to distinguish disease status. Model performance was assessed using 5-fold cross-validation, in which out-of-fold prediction probabilities were aggregated across all samples and used to compute receiver operating characteristic (ROC) curves, AUC, and confusion matrices. Given the sum-to-one constraint of factor composition, inclusion of the top two factors effectively captures the remaining compositional variation represented by the third factor. In addition, we calculated the Shannon entropy of each sample’s factor composition to quantify ecological complexity and heterogeneity. Together, this framework enables systematic identification of ecologically robust and clinically informative factors.

**4.2 Correlation between MEFs and taxa**

To examine the association between Wiredancer-derived ecological factors and disease-related microbial shifts, we calculated species-level t-statistics based on log-transformed relative abundances between healthy and disease groups. These values served as effect size estimates reflecting the direction and magnitude of taxonomic differences. Pearson correlations were then computed between the t-statistics and taxon loadings from the factor-by-taxon matrix to assess the alignment between ecological factor structure and species-level variation.

**5.1 Intra-individual temporal stability**

Cosine similarity was calculated between all available timepoints for each subject. MEFs values were row-normalized such that the three components summed to one per sample. Cosine similarity quantifies the similarity in MEFs composition between visits, with higher values indicating more stable ecological profiles. For each subject with repeated sampling, pairwise similarity scores were computed across timepoints. Group-level comparisons were conducted using Wilcoxon rank-sum tests to examine differences in MEFs stability between healthy and disease groups. Linear regression was further used to evaluate whether temporal similarity decayed as a function of the time interval between visits. This analysis provided a measure of how consistent MEF-defined ecological configurations were within individuals and how disease states might impact ecological fluctuation over time.

**5.2 MEFs state transitions**

We used a discrete-time Markov chain model to characterize transitions between MEF-defined ecological states over time. Each sample was assigned an MEF state based on its classification, and only within-subject transitions were considered to ensure biological relevance. Sample timepoints were ordered chronologically, and transitions were extracted from longitudinal trajectories. Modeling was performed using the markovchain package in R.

**5.3 Compositional modeling of MEF3 based on MEF1 and MEF2**

We used the normalized individual-level factor score matrix for downstream analyses to ensure comparability across samples, and further applied CLR transformation to account for the dependency induced by the constant-sum constraint among components, without altering the underlying data structure. To evaluate the dependency of MEF3 on MEF1 and MEF2, we constructed a linear mixed-effects model using CLR transformed MEFs values. The model included MEF1 and MEF2 as fixed effects and subject identity as a random intercept to account for repeated measures. Only individuals with at least three timepoints were included to ensure model robustness. The goal was to assess whether MEF3 could be consistently inferred from the other two components across subjects and timepoints under compositional constraints. Model fit and fixed effect significance were evaluated using the lme4 package in R.

**Supplemental Figures**

**Figure S1.** Microbial diversity and its association with individual-level characteristics. a) R^2^ values from PERMANOVA analyses indicate the proportion of variance in microbiome composition explained by various covariates (n = 6,790). b) Violin and boxplots illustrate differences in Simpson diversity indices between healthy and unhealthy groups; **** indicates statistical significance (n = 20,178; *p* < 0.0001, Wilcoxon rank-sum test). c-e) Comparisons of Shannon diversity across sex (c), age group (d; < 60 vs ≥ 60 years), and BMI category (e; < 25 vs ≥ 25). Group distributions are shown using scatter and boxplots, and *p*-values were calculated using Wilcoxon tests (n = 6,790). Only the BMI-based comparison showed no significant difference (ns).

**Figure S2.** Abundance differences of representative microbial species between Healthy and Unhealthy groups. Each subplot displays the association results for a representative species, including the regression coefficient (β), false discovery rate (FDR)-adjusted *p*-value, and direction of change. Positive coefficients indicate higher relative abundance in the Unhealthy group, whereas negative coefficients indicate enrichment in the Healthy group (n = 20,178).

**Figure S3.** Clustering stability analysis of microbial features using PAM across different subsampling levels (n = 11,823). a) Calinski–Harabasz (CH) index curves for k = 2 to 10 clusters under subsampling proportions of 5%, 25%, 50%, 75%, and 100%. Higher CH scores consistently support k = 2 as the optimal cluster number across sampling levels. b) Frequency of the best-performing cluster number (k) based on Silhouette scores over 20 iterations, with k = 2 being most frequently selected. c) Mean ± standard error of silhouette scores for each k value, showing consistently higher clustering quality at k = 2.

**Figure S4.** DMM-based model selection for determining the optimal number of clusters across varying sample sizes (n = 11,823). a-e) Model fitting results based on DMM modeling using 5%, 25%, 50%, 75%, and 100% of the data. Model performance metrics (AIC and BIC) are plotted against the number of clusters (k).

**Figure S5.** Model stability evaluation based on cohort-stratified cross-validation. The cophenetic correlation coefficient was used to assess decomposition stability across different numbers of factors (n = 11,823).

**Figure S6.** Correlation among MEFs components. Heatmap showing pairwise Spearman correlations between the three MEFs, based on the row-wise normalized H matrix from the decomposition model. Hierarchical clustering was applied to both rows and columns. Color scale represents correlation strength from low (blue) to high (red) (n = 11,823).

**Figure S7.** Significantly enriched metabolic pathways associated with MEFs. Bar plots depict the top 10 enriched pathways for each microbial ecological factor (MEF1-3) after normalization of pathway scores. Pathway scores were calculated as the product of species-level factor weights and the mean pathway abundances inferred from the MetaCyc database (n = 11,823).

**Figure S8.** Distribution of t-statistics for identifying differential microbial features. The density plot illustrates the distribution of raw t-statistics derived from between-group comparisons of microbial abundances (n = 18,873).

**Figure S9.** Differential distribution of MEFs between disease groups and healthy controls. Boxplots show the distributions of three microbial ecological factors (MEF1 in blue, MEF2 in yellow, MEF3 in pink) across multiple disease cohorts compared with healthy controls. Statistical significance was assessed using the two-sided Wilcoxon rank-sum test, with *p*-values shown above each comparison. Disease abbreviations are as follows: ACVD (Atherosclerotic Cardiovascular Disease), CAD (Coronary Artery Disease), T2D (Type 2 Diabetes), CRC (Colorectal Cancer), HF (Heart Failure), IBD (Inflammatory Bowel Disease), IBS (Irritable Bowel Syndrome), IGT (Impaired Glucose Tolerance), MS (Metabolic Syndrome), ME/CFS (Myalgic Encephalomyelitis/Chronic Fatigue Syndrome), PD (Parkinson’s Disease), RA (Rheumatoid Arthritis), STEC (Shiga toxin-producing E. coli infection), STH (Soil-Transmitted Helminth infection), and T1D (Type 1 Diabetes). Groups with fewer than 20 samples were excluded (n = 18,873).

**Figure S10.** Distributions of MEFs scores across sex, age, and BMI groups. Violin plots illustrate the distributions of MEF1, MEF2, and MEF3 stratified by sex (female vs male; a, d, g), age group (< 60 vs ≥ 60 years; b, e, h), and BMI category (≥ 25 vs < 25; c, f, i). *p*-values are derived from Wilcoxon rank-sum tests (n = 6,790).

**Figure S11.** ROC curves of logistic regression models based on Shannon entropy and MEFs factors (n = 18,873). a) ROC curves of single-factor logistic regression models based on MEF1, MEF2, and MEF3. The AUC reflects the predictive performance of each model in distinguishing healthy and unhealthy individuals. b) ROC curve of a univariable logistic regression model constructed using Shannon entropy.

**Figure S12.** Effect sizes (Cohen’s *d*) of cognitive differences between SZ and healthy controls. The radar plot shows cognitive deficits in SZ across five domains. Negative values indicate lower performance; asterisks denote significant differences (n = 106).

**Figure S13.** Association between MEFs and cognition after covariate adjustment. After residualizing for age, sex, BMI, and years of education using linear models, the CCA-derived associations remained statistically significant (n = 471).

**Figure S14.** Correlation analysis between MEFs factors, clinical scales, and blood biomarkers (n = 230). a) Spearman correlation between MEF1-3 and clinical subscales, including HAMD-depression, HAMA-anxiety, and HAMD-suicide scores. b) Heatmap showing Spearman correlations between MEFs factors and 35 blood biomarkers. Statistical significance is based on BH-adjusted *p*-values and indicated by asterisks.

**Figure S15.** Selection of the optimal number of clusters for MDD samples based on MEF-derived features using k-means clustering. The plot shows the average silhouette width for different cluster numbers (K = 2-10). The highest silhouette score is observed at K = 2, indicating the optimal clustering solution (n = 230).

**Figure S16.** Longitudinal changes in MEF scores from baseline to follow-up. Paired dot plots show individual-level changes in MEF1, MEF2, and MEF3 scores over time. Each line connects scores from the same subject at baseline and follow-up, reflecting within-subject variation during the observation period (n = 42).

**Figure S17.** Consistency before and after applying a 0.01% abundance filtering threshold (n = 20,178). a) Shannon diversity shows high concordance between raw data and 0.01% filtered data (Spearman r = 0.998). b) The distribution of pairwise cosine similarity is largely overlapping between the two datasets (Spearman r = 0.997), indicating minimal impact of filtering on the overall structure.

**Figure S18**. Robustness across different prevalence thresholds (n = 20,178). a) The distribution of Shannon diversity is highly similar across prevalence thresholds (5%, 10%, 15%, and 20%). b-d) Shannon diversity shows strong correlations between each threshold (5%, 15%, 20%) and the baseline (10%), indicating high within-sample consistency. e) The distributions of pairwise cosine similarity largely overlap across thresholds, suggesting minimal impact on the overall similarity structure. f) Pairwise cosine similarity matrices across thresholds remain highly consistent with the baseline (10%), with Spearman correlation coefficients exceeding 0.97, indicating robust sample-level structure.

**Figure S19.** Comparison of explained variance across methods. MEF shows higher explained variance compared to PCA and RDA, indicating improved ability to capture underlying data structure (n = 18,873).

**Figure S20.** Comparison of different factor-learning strategies (n = 18,873). a) Comparison of explained variance across factor learning strategies. b-c) Correspondence of MEFs derived from different strategies. d-f) PCA projections of samples based on the healthy-only, all-sample, and split models. g-h) ROC curves based on factor representations from the all-sample model and the split-test model. i) Bootstrap AUC estimates with 95% confidence intervals. j-k) Permutation test results for model discrimination performance. l) ICC-based stability assessment across different random seeds.

**Figure S21.** Robustness analysis across cohorts and preprocessing strategies (n = 20,178). a) Mean explained variance across cohorts, showing consistent model performance. b) Explained variance under different preprocessing strategies (ComBat, log1p, and square-root transformation). c) Proportion of variance explained by cohort identity (R²) for the top three MEFs. d) Bootstrap-based stability of MEFs, measured by mean Spearman correlation.

**Figure S22.** Cross-cohort variability analysis in MEF space (n = 20,178). a) Global distribution of healthy and disease samples in MEF space. b) Comparison of distances to group centroid between healthy and disease samples within each cohort. c) Cross-cohort comparison of distance differences (disease vs healthy). d) Variability among healthy samples across cohorts (healthy-healthy comparison). e) Within-cohort comparison of dispersion differences between healthy and disease groups. f) Comparison of distances to centroid between healthy and disease samples after covariate adjustment. g) Multivariable regression analysis of factors associated with dispersion (effect size with 95% CI).

**Table S1.** Summary of cohort information.

| **study_name** | **sample_count** |
| --- | --- |
| MetaCardis_2020_a | 1831 |
| LifeLinesDeep_2016 | 1135 |
| AsnicarF_2021 | 1098 |
| MehtaRS_2018 | 928 |
| ZeeviD_2015 | 900 |
| LloydPriceJ_2019 | 848 |
| YachidaS_2019 | 616 |
| Zhu_2025 | 609 |
| Mars_2020 | 509 |
| SchirmerM_2016 | 456 |
| JieZ_2017 | 385 |
| RenallN_2023 | 382 |
| TeeM_2022 | 375 |
| VilaAV_2018 | 355 |
| KangJ_2022 | 352 |
| QinJ_2012 | 342 |
| Yang_2020 | 311 |
| HMP_2019_t2d | 296 |
| LeChatelierE_2013 | 292 |
| LiJ_2014 | 260 |
| HallAB_2017 | 259 |
| Yeoh_2021 | 255 |
| CosteaPI_2017 | 253 |
| XieH_2016 | 250 |
| Liu_2022 | 243 |
| QinN_2014 | 237 |
| NielsenHB_2014 | 219 |
| HansenLBS_2018 | 207 |
| VincentC_2016 | 200 |
| Zhang_2022 | 199 |
| LiJ_2017 | 196 |
| Ghosh_et_al_2020 | 188 |
| ShaoY_2019 | 174 |
| LeeKA_2022 | 165 |
| ZhuF_2020 | 162 |
| RubelMA_2020 | 156 |
| ZellerG_2014 | 156 |
| BritoIL_2016 | 154 |
| FengQ_2015 | 154 |
| Li_2020 | 149 |
| HMP_2012 | 147 |
| KarlssonFH_2013 | 138 |
| Jeffery_et_al_2020 | 136 |
| YuJ_2015 | 128 |
| WirbelJ_2018 | 125 |
| PehrssonE_2016 | 120 |
| KeohaneDM_2020 | 117 |
| YassourM_2018 | 116 |
| PasolliE_2019 | 111 |
| LiuW_2016 | 110 |
| VogtmannE_2016 | 104 |
| MobegiF_2020 | 100 |
| NagySzakalD_2017 | 100 |
| BackhedF_2015 | 99 |
| DeFilippisF_2019 | 97 |
| ChengpingW_2017 | 95 |
| LouisS_2016 | 92 |
| DhakanDB_2019 | 88 |
| LoombaR_2017 | 86 |
| Barton_et_al_2018 | 84 |
| HanniganGD_2017 | 81 |
| ThomasAM_2018a | 80 |
| ThomasAM_2019_c | 80 |
| Kovtun_2022 | 74 |
| RaymondF_2016 | 72 |
| Bengtsson-PalmeJ_2015 | 70 |
| Zuo_2020 | 67 |
| Li_2021 | 65 |
| YeZ_2018 | 65 |
| GuptaA_2019 | 60 |
| ThomasAM_2018b | 60 |
| BedarfJR_2017 | 58 |
| LokmerA_2019 | 57 |
| PallejaA_2018 | 57 |
| Cronin_et_al_2018 | 55 |
| LiSS_2016 | 55 |
| LomanNJ_2013 | 42 |
| FrankelAE_2017 | 39 |
| MatsonV_2018 | 39 |
| TettAJ_2019_a | 38 |
| Obregon-TitoAJ_2015 | 37 |
| SankaranarayananK_2015 | 37 |
| YassourM_2016 | 36 |
| Heitz-BuschartA_2016 | 35 |
| DavidLA_2015 | 33 |
| RampelliS_2015 | 33 |
| KaurK_2020 | 31 |
| SmitsSA_2017 | 31 |
| PetersBA_2019 | 27 |
| GopalakrishnanV_2018 | 25 |
| IjazUZ_2017 | 25 |
| TettAJ_2019_c | 24 |
| TettAJ_2019_b | 23 |
| FerrettiP_2018 | 22 |
| RosaBA_2018 | 20 |
| WindTT_2020 | 20 |
| ChuDM_2017 | 17 |
| HMP_2019_ibdmdb | 17 |
| WampachL_2018 | 14 |
| AsnicarF_2017 | 8 |

**Table S2.** Batch effect correction comparison.

| Before adjust | | | After adjust | | |
| --- | --- | --- | --- | --- | --- |
| **Factor** | **R^2** | ***p*** | **Factor** | **R^2** | ***p*** |
| Cohort | 0.20608 | < 0.001 | Cohort | 0.06658 | < 0.001 |
| BMI | 0.00344 | < 0.001 | BMI | 0.00155 | < 0.001 |
| Age | 0.00401 | < 0.001 | Age | 0.00271 | < 0.001 |
| Gender | 0.00425 | < 0.001 | Gender | 0.00315 | < 0.001 |
| Diseasestatus | 0.01698 | < 0.001 | Diseasestatus | 0.00701 | < 0.001 |
| Country | 0.1658 | < 0.001 | Country | 0.05002 | < 0.001 |
| Sequencing platform | 0.002472 | < 0.001 | Sequencing platform | 0.000803 | < 0.001 |

**Table S3.** Differential species analysis.

| **feature** | **metadata** | **value** | **coef** | **stderr** | **pval** | **qval** |
| --- | --- | --- | --- | --- | --- | --- |
| *Roseburia_sp_CAG_471* | study_condition | Unhealthy | -2.5353 | 0.0781788642253403 | 6.11502387300903e-225 | 1.22300477460181e-222 |
| *Firmicutes_bacterium_CAG_110* | study_condition | Unhealthy | -2.40984 | 0.0769094530308379 | 1.77155441778654e-210 | 1.77155441778654e-208 |
| *Oscillibacter_sp_CAG_241* | study_condition | Unhealthy | -2.73091 | 0.0888101623301948 | 5.80500980433492e-203 | 3.87000653622328e-201 |
| *Oscillibacter_sp_57_20* | study_condition | Unhealthy | -3.14577 | 0.102408960509621 | 1.49971404472021e-202 | 7.49857022360103e-201 |
| *Eubacterium_hallii* | study_condition | Unhealthy | -3.33368 | 0.111203826259876 | 3.22012500073242e-193 | 1.28805000029297e-191 |
| *Firmicutes_bacterium_CAG_83* | study_condition | Unhealthy | -2.51154 | 0.0876938182276234 | 7.33273122329186e-177 | 2.44424374109729e-175 |
| *Hungatella_hathewayi* | study_condition | Unhealthy | 1.75791503986198 | 0.0628438973328021 | 5.79423297864357e-169 | 1.6554951367553e-167 |
| *Asaccharobacter_celatus* | study_condition | Unhealthy | -2.32713 | 0.0835140664319223 | 1.04942195108128e-167 | 2.62355487770321e-166 |
| *Coprococcus_comes* | study_condition | Unhealthy | -2.59818 | 0.0941556090599872 | 1.45973642437966e-164 | 3.24385872084368e-163 |
| *Eubacterium_eligens* | study_condition | Unhealthy | -2.95878 | 0.107523966634601 | 1.13321745372457e-163 | 2.26643490744914e-162 |
| *Erysipelatoclostridium_ramosum* | study_condition | Unhealthy | 1.80838779975708 | 0.0658089914722759 | 3.11388762704165e-163 | 5.66161386734846e-162 |
| *Coprococcus_catus* | study_condition | Unhealthy | -2.05073 | 0.0748567294200234 | 2.88121730916224e-162 | 4.80202884860373e-161 |
| *Coprococcus_eutactus* | study_condition | Unhealthy | -2.83166 | 0.103657227151067 | 2.2487704019629e-161 | 3.45964677225062e-160 |
| *Gemmiger_formicilis* | study_condition | Unhealthy | -2.24911 | 0.0845518391906748 | 2.93762371340276e-153 | 4.19660530486109e-152 |
| *Clostridium_bolteae* | study_condition | Unhealthy | 1.79158348640266 | 0.0676927414101222 | 9.15401276503587e-152 | 1.22053503533812e-150 |
| *Ruminococcus_gnavus* | study_condition | Unhealthy | 2.3246 | 0.0890567017169007 | 9.63213200661571e-148 | 1.20401650082696e-146 |
| *Clostridium_symbiosum* | study_condition | Unhealthy | 1.83737351460938 | 0.0706678846397838 | 1.27695674526668e-146 | 1.50230205325492e-145 |
| *Romboutsia_ilealis* | study_condition | Unhealthy | -1.2117 | 0.0476976869973553 | 3.65384334445284e-140 | 4.05982593828093e-139 |
| *Faecalibacterium_prausnitzii* | study_condition | Unhealthy | -1.7692 | 0.0710254579401404 | 6.40138109931603e-135 | 6.73829589401687e-134 |
| *Roseburia_sp_CAG_182* | study_condition | Unhealthy | -1.4014 | 0.056565643898799 | 1.65631614346896e-133 | 1.65631614346896e-132 |
| *Firmicutes_bacterium_CAG_95* | study_condition | Unhealthy | -1.58867 | 0.0649501421914265 | 3.10674077115856e-130 | 2.95880073443672e-129 |
| *Bifidobacterium_adolescentis* | study_condition | Unhealthy | -2.82565 | 0.116754716615099 | 1.40547982362316e-127 | 1.27770893056651e-126 |
| *Adlercreutzia_equolifaciens* | study_condition | Unhealthy | -1.90531 | 0.0802616451507753 | 6.93610562607575e-123 | 6.03139619658761e-122 |
| *Eubacterium_ramulus* | study_condition | Unhealthy | -1.68559 | 0.0714104770172653 | 1.5433107311248e-121 | 1.28609227593733e-120 |
| *Clostridium_clostridioforme* | study_condition | Unhealthy | 1.24104719335307 | 0.052915137652992 | 4.89992723703628e-120 | 3.91994178962902e-119 |
| *Clostridium_sp_CAG_167* | study_condition | Unhealthy | -1.4347 | 0.061972575827845 | 4.80567281895219e-117 | 3.696671399194e-116 |
| *Roseburia_hominis* | study_condition | Unhealthy | -1.863 | 0.0808756523350393 | 6.44459033181974e-116 | 4.77377061616277e-115 |
| *Eubacterium_sp_CAG_38* | study_condition | Unhealthy | -1.91493 | 0.0847347379771976 | 1.07421294936266e-111 | 7.67294963830472e-111 |
| *Dorea_formicigenerans* | study_condition | Unhealthy | -1.58483 | 0.0729511899234308 | 1.84388534790495e-103 | 1.27164506752066e-102 |
| *Barnesiella_intestinihominis* | study_condition | Unhealthy | -2.39455 | 0.111185076182183 | 9.87518946264729e-102 | 6.58345964176486e-101 |
| *Dorea_longicatena* | study_condition | Unhealthy | -1.75804 | 0.0823245698685062 | 4.48994162962371e-100 | 2.8967365352411e-99 |
| *Clostridium_scindens* | study_condition | Unhealthy | 0.890804275417124 | 0.0427756147452234 | 2.58122397734238e-95 | 1.61326498583899e-94 |
| *Eubacterium_rectale* | study_condition | Unhealthy | -1.72232 | 0.0827381757140229 | 3.07459793117694e-95 | 1.86339268556178e-94 |
| *Eubacterium_siraeum* | study_condition | Unhealthy | -1.91683 | 0.0944836843998056 | 1.33034867900595e-90 | 7.82558046474088e-90 |
| *Ruminococcus_torques* | study_condition | Unhealthy | -2.0613 | 0.101803193320129 | 2.92413309108585e-90 | 1.6709331949062e-89 |
| *Bifidobacterium_catenulatum* | study_condition | Unhealthy | -1.20643 | 0.059801027806356 | 1.26791339407145e-89 | 7.04396330039697e-89 |
| *Enterorhabdus_caecimuris* | study_condition | Unhealthy | -1.2042 | 0.0600619289336934 | 1.49159390449066e-88 | 8.06266975400358e-88 |
| *Slackia_isoflavoniconvertens* | study_condition | Unhealthy | -1.9656 | 0.0984987163177886 | 9.41956395245293e-88 | 4.95766523813312e-87 |
| *Olsenella_scatoligenes* | study_condition | Unhealthy | -1.11289 | 0.0562584475606683 | 2.80898913811021e-86 | 1.44050725031293e-85 |
| *Firmicutes_bacterium_CAG_170* | study_condition | Unhealthy | -1.22309 | 0.062713116964039 | 6.14043581716877e-84 | 3.07021790858438e-83 |
| *Clostridium_aldenense* | study_condition | Unhealthy | 0.652984605906253 | 0.0335565597092082 | 1.42072196269159e-83 | 6.93035103751995e-83 |
| *Ruminococcus_bicirculans* | study_condition | Unhealthy | -1.99203 | 0.102777382086033 | 6.21690442132889e-83 | 2.96043067682328e-82 |
| *Agathobaculum_butyriciproducens* | study_condition | Unhealthy | -1.37642 | 0.0714312137423612 | 5.31608676077281e-82 | 2.4725984933827e-81 |
| *Flavonifractor_plautii* | study_condition | Unhealthy | 1.45651676253076 | 0.0758354573379228 | 1.75066206920747e-81 | 7.95755486003395e-81 |
| *Roseburia_sp_CAG_309* | study_condition | Unhealthy | -0.91066 | 0.0476898414625451 | 1.41555413402881e-80 | 6.2913517067947e-80 |
| *Ruminococcus_lactaris* | study_condition | Unhealthy | -1.91541 | 0.1035827981666 | 1.02044198157898e-75 | 4.43670426773471e-75 |
| *Roseburia_intestinalis* | study_condition | Unhealthy | -1.7626 | 0.0956301075613708 | 3.04793872203894e-75 | 1.29699520086763e-74 |
| *Fusicatenibacter_saccharivorans* | study_condition | Unhealthy | -1.50529 | 0.0820055945294375 | 1.1981991460117e-74 | 4.99249644171543e-74 |
| *Butyrivibrio_crossotus* | study_condition | Unhealthy | -1.44173 | 0.0791175545269573 | 1.32992975503619e-73 | 5.42828471443344e-73 |
| *Lactobacillus_rogosae* | study_condition | Unhealthy | -0.99049 | 0.0547889261793365 | 1.76399101697924e-72 | 7.05596406791695e-72 |
| *Streptococcus_oralis* | study_condition | Unhealthy | 0.832981562498796 | 0.0463175593329661 | 9.42648183585974e-72 | 3.69665954347441e-71 |
| *Mogibacterium_diversum* | study_condition | Unhealthy | 0.674900376070514 | 0.0376930697791232 | 3.82024455651747e-71 | 1.46932482942979e-70 |
| *Roseburia_faecis* | study_condition | Unhealthy | -1.78334 | 0.10064625079251 | 1.01111071566931e-69 | 3.81551213460119e-69 |
| *Roseburia_inulinivorans* | study_condition | Unhealthy | -1.37672 | 0.0784819204241553 | 2.21136523975769e-68 | 8.19024162873218e-68 |
| *Collinsella_aerofaciens* | study_condition | Unhealthy | -2.03735 | 0.117189560899808 | 3.30807682122581e-67 | 1.20293702590029e-66 |
| *Methanobrevibacter_smithii* | study_condition | Unhealthy | -1.77228 | 0.102446750309609 | 1.43289462579637e-66 | 5.11748080641559e-66 |
| *Streptococcus_anginosus_group* | study_condition | Unhealthy | 0.745613383445585 | 0.0435245527494316 | 2.53138806729793e-65 | 8.88206339402781e-65 |
| *Lachnospira_pectinoschiza* | study_condition | Unhealthy | -1.89712 | 0.11138404443248 | 1.33933406090386e-64 | 4.61839331346159e-64 |
| *Holdemanella_biformis* | study_condition | Unhealthy | -1.72275 | 0.101640676825603 | 5.39173387592499e-64 | 1.82770639861864e-63 |
| *Odoribacter_splanchnicus* | study_condition | Unhealthy | -1.60921 | 0.0955436479753249 | 3.21191021679585e-63 | 1.07063673893195e-62 |
| *Eubacterium_sp_CAG_251* | study_condition | Unhealthy | -1.4868 | 0.0892914910142083 | 7.68277116817055e-62 | 2.5189413666133e-61 |
| *Eubacterium_ventriosum* | study_condition | Unhealthy | -1.40612 | 0.084969890150453 | 4.1549507623955e-61 | 1.34030669754693e-60 |
| *Eisenbergiella_massiliensis* | study_condition | Unhealthy | 1.20578098762818 | 0.0729255615165197 | 5.22809088695273e-61 | 1.65971139268341e-60 |
| *Blautia_faecis* | study_condition | Unhealthy | 0.763698614936772 | 0.0462752090756901 | 8.68112714529233e-61 | 2.71285223290385e-60 |
| *Collinsella_intestinalis* | study_condition | Unhealthy | -1.10221 | 0.0669652578920957 | 1.77901717428176e-60 | 5.47389899779005e-60 |
| *Blautia_obeum* | study_condition | Unhealthy | -1.43163 | 0.0871430256410743 | 2.94480819564405e-60 | 8.92366119892137e-60 |
| *Alistipes_shahii* | study_condition | Unhealthy | -1.52634 | 0.0940607164498465 | 7.64361229714701e-59 | 2.2816753125812e-58 |
| *Enorma_massiliensis* | study_condition | Unhealthy | -0.96712 | 0.0597832387553911 | 1.70911890154433e-58 | 5.02682029865981e-58 |
| *Aeriscardovia_aeriphila* | study_condition | Unhealthy | -0.42041 | 0.0265156799169617 | 2.83169602925716e-56 | 8.20781457755698e-56 |
| *Ruminococcus_bromii* | study_condition | Unhealthy | -2.19037 | 0.139006425438962 | 1.31320808164839e-55 | 3.75202309042396e-55 |
| *Streptococcus_vestibularis* | study_condition | Unhealthy | 0.668909259812168 | 0.0425194239704427 | 1.95429554186428e-55 | 5.50505786440643e-55 |
| *Streptococcus_gordonii* | study_condition | Unhealthy | 0.736195800910665 | 0.0474061099272265 | 4.50112225019949e-54 | 1.25031173616653e-53 |
| *Prevotella_copri* | study_condition | Unhealthy | -2.3426 | 0.152566437274106 | 6.5679216166465e-53 | 1.79943057990315e-52 |
| *Turicibacter_sanguinis* | study_condition | Unhealthy | -0.86804 | 0.0571181770363447 | 7.13695936626499e-52 | 1.92890793682838e-51 |
| *Klebsiella_pneumoniae* | study_condition | Unhealthy | 1.01212715232875 | 0.0679242893789187 | 6.00357515965117e-50 | 1.60095337590698e-49 |
| *Sellimonas_intestinalis* | study_condition | Unhealthy | 0.874092341309313 | 0.0590542171444118 | 2.59856192785958e-49 | 6.83832086278836e-49 |
| *Clostridium_disporicum* | study_condition | Unhealthy | -0.77743 | 0.0526098236317564 | 3.7144991152046e-49 | 9.64804964988208e-49 |
| *Clostridium_citroniae* | study_condition | Unhealthy | 0.91889418652193 | 0.0628985420216142 | 4.32371942949174e-48 | 1.10864600756199e-47 |
| *Escherichia_coli* | study_condition | Unhealthy | 1.3624316316932 | 0.0933215207891522 | 4.98625263535027e-48 | 1.26234243932918e-47 |
| *Bacteroides_galacturonicus* | study_condition | Unhealthy | -0.70663 | 0.0485979499850714 | 1.17186160003347e-47 | 2.92965400008368e-47 |
| *Eggerthella_lenta* | study_condition | Unhealthy | 1.17715849708386 | 0.0812741888604612 | 2.6499735411068e-47 | 6.54314454594271e-47 |
| *Bifidobacterium_dentium* | study_condition | Unhealthy | 0.813594070011763 | 0.0569544164571537 | 4.55369729938487e-46 | 1.11065787789875e-45 |
| *Clostridium_sp_CAG_253* | study_condition | Unhealthy | -0.616 | 0.0431514166272989 | 5.22059134754709e-46 | 1.25797381868605e-45 |
| *Clostridium_lavalense* | study_condition | Unhealthy | 0.696689081407409 | 0.0494589331971509 | 7.53239199644046e-45 | 1.79342666581916e-44 |
| *Phocaeicola_vulgatus* | study_condition | Unhealthy | 0.710161959859864 | 0.0504835174937136 | 9.83076200572136e-45 | 2.31312047193444e-44 |
| *Clostridium_innocuum* | study_condition | Unhealthy | 0.936007238399068 | 0.0667334821995562 | 1.7476450121527e-44 | 4.06429072593652e-44 |
| *Ruminococcus_callidus* | study_condition | Unhealthy | -0.83672 | 0.0597682977930396 | 2.53259017281622e-44 | 5.82204637429016e-44 |
| *Alistipes_putredinis* | study_condition | Unhealthy | -1.54199 | 0.110848742851538 | 8.6823109909644e-44 | 1.97325249794645e-43 |
| *Anaerostipes_hadrus* | study_condition | Unhealthy | -0.9575 | 0.069265317883956 | 2.88842413098317e-43 | 6.49084074378242e-43 |
| *Clostridium_bolteae_CAG_59* | study_condition | Unhealthy | 0.537583499943106 | 0.0389049670476011 | 3.1303803774343e-43 | 6.95640083874289e-43 |
| *Clostridiaceae_bacterium* | study_condition | Unhealthy | 0.735053164195525 | 0.0542036424584946 | 1.03969846883195e-41 | 2.28505157985043e-41 |
| *Blautia_sp_CAG_257* | study_condition | Unhealthy | 0.763919418117781 | 0.0573193609327127 | 2.37532776751824e-40 | 5.163756016344e-40 |
| *Catenibacterium_mitsuokai* | study_condition | Unhealthy | -0.81714 | 0.0616978437588933 | 7.14873236859536e-40 | 1.53736179969793e-39 |
| *Anaerotignum_lactatifermentans* | study_condition | Unhealthy | 0.618584977854456 | 0.0476383429569497 | 2.12093794406402e-38 | 4.51263392354047e-38 |
| *Gemella_sanguinis* | study_condition | Unhealthy | 0.481976202715847 | 0.0374216552948178 | 8.26406499016543e-38 | 1.7398031558243e-37 |
| *Bacteroides_vulgatus* | study_condition | Unhealthy | -1.60685 | 0.12608932099391 | 4.69358918960194e-37 | 9.77831081167071e-37 |
| *Alistipes_finegoldii* | study_condition | Unhealthy | -1.25333 | 0.0987921224527619 | 9.70525216641752e-37 | 2.00108292091083e-36 |
| *Anaerotruncus_colihominis* | study_condition | Unhealthy | 0.803878998889763 | 0.0639051681629265 | 3.75389083714668e-36 | 7.66100170846261e-36 |
| *Klebsiella_quasipneumoniae* | study_condition | Unhealthy | 0.564922466303606 | 0.0456483511583124 | 4.75234445535074e-35 | 9.60069586939544e-35 |
| *Streptococcus_mitis* | study_condition | Unhealthy | 0.600887740433449 | 0.0493732415197465 | 5.8848348083104e-34 | 1.17696696166208e-33 |
| *Klebsiella_variicola* | study_condition | Unhealthy | 0.546887241962921 | 0.0453285695321182 | 2.10799340258188e-33 | 4.17424436154828e-33 |
| *Firmicutes_bacterium_CAG_238* | study_condition | Unhealthy | -0.55902 | 0.0470496128797736 | 1.89384540412721e-32 | 3.71342236103374e-32 |
| *Streptococcus_sanguinis* | study_condition | Unhealthy | 0.456503906777745 | 0.0386287955132136 | 4.03564609004983e-32 | 7.83620600009675e-32 |
| *Intestinibacter_bartlettii* | study_condition | Unhealthy | -0.9789 | 0.0833854555700385 | 1.01458367923477e-31 | 1.95112246006687e-31 |
| *Akkermansia_muciniphila* | study_condition | Unhealthy | -1.1802 | 0.104563416903601 | 1.86563119645689e-29 | 3.55358323134645e-29 |
| *Veillonella_parvula* | study_condition | Unhealthy | 0.846724031813273 | 0.0750936022259906 | 2.12130995807366e-29 | 4.00247161900691e-29 |
| *Streptococcus_infantis* | study_condition | Unhealthy | 0.393272085835396 | 0.0349288594550854 | 2.55112191792138e-29 | 4.76845218303061e-29 |
| *Clostridium_asparagiforme* | study_condition | Unhealthy | 0.489945239637211 | 0.0440721046454097 | 1.2573005009398e-28 | 2.32833426099963e-28 |
| *Eisenbergiella_tayi* | study_condition | Unhealthy | 0.642027245148119 | 0.0604320002582127 | 2.70581192121473e-26 | 4.96479251599033e-26 |
| *Phascolarctobacterium_faecium* | study_condition | Unhealthy | 1.00485068024105 | 0.0962752277003751 | 1.94518542146776e-25 | 3.53670076630502e-25 |
| *Bifidobacterium_longum* | study_condition | Unhealthy | -1.04131 | 0.102085168046999 | 2.26280297348853e-24 | 4.0771224747541e-24 |
| *Clostridium_saccharolyticum* | study_condition | Unhealthy | 0.451335167853656 | 0.0443048937268266 | 2.59477028359455e-24 | 4.63351836356169e-24 |
| *Victivallis_vadensis* | study_condition | Unhealthy | -0.46872 | 0.0460645165624267 | 2.92742046383371e-24 | 5.1812751572278e-24 |
| *Streptococcus_thermophilus* | study_condition | Unhealthy | -0.70223 | 0.0698950461509877 | 1.07800085155002e-23 | 1.89122956412285e-23 |
| *Bacteroides_fragilis* | study_condition | Unhealthy | 0.918220255129022 | 0.092982781697876 | 6.01382648697572e-23 | 1.04588286730013e-22 |
| *Bacteroides_sp_CAG_144* | study_condition | Unhealthy | -0.62814 | 0.0662518965326376 | 2.78701274404943e-21 | 4.80519438629212e-21 |
| *Alistipes_inops* | study_condition | Unhealthy | -0.72288 | 0.0782017290697819 | 2.6095267044245e-20 | 4.46072940927265e-20 |
| *Blautia_wexlerae* | study_condition | Unhealthy | -0.73578 | 0.0803739428402373 | 5.97321943599025e-20 | 1.01241007389665e-19 |
| *Coprobacter_fastidiosus* | study_condition | Unhealthy | -0.73561 | 0.0823934538080094 | 4.70255672575904e-19 | 7.90345668194796e-19 |
| *Eubacterium_sp_CAG_274* | study_condition | Unhealthy | -0.56018 | 0.0637386693093013 | 1.63108079175636e-18 | 2.7184679862606e-18 |
| *Dielma_fastidiosa* | study_condition | Unhealthy | 0.414485631215316 | 0.0473058892291254 | 2.0703238965896e-18 | 3.42202296956959e-18 |
| *Desulfovibrio_piger* | study_condition | Unhealthy | -0.76254 | 0.0883708798161496 | 6.64692629389281e-18 | 1.08966004817915e-17 |
| *Actinomyces_sp_HMSC035G02* | study_condition | Unhealthy | 0.298106595133481 | 0.0345991766264839 | 7.4355149781099e-18 | 1.20902682570893e-17 |
| *Blautia_hydrogenotrophica* | study_condition | Unhealthy | 0.408056755918608 | 0.0474718801246762 | 8.86529430348119e-18 | 1.42988617798084e-17 |
| *Veillonella_atypica* | study_condition | Unhealthy | 0.471594506823208 | 0.0553061530018482 | 1.60648134622434e-17 | 2.57037015395895e-17 |
| *Prevotella_sp_CAG_279* | study_condition | Unhealthy | -0.55353 | 0.066090355750793 | 5.86166965917401e-17 | 9.30423755424446e-17 |
| *Alistipes_indistinctus* | study_condition | Unhealthy | -0.68464 | 0.0825186924573059 | 1.1365478845862e-16 | 1.78983918832473e-16 |
| *Bacteroides_dorei* | study_condition | Unhealthy | -0.97691 | 0.119911019362509 | 3.94679965492788e-16 | 6.16687446082481e-16 |
| *Paraprevotella_xylaniphila* | study_condition | Unhealthy | -0.66098 | 0.0812641532948177 | 4.39955423139674e-16 | 6.82101431224301e-16 |
| *Coprobacter_secundus* | study_condition | Unhealthy | -0.46446 | 0.0577271886917794 | 9.03995261019966e-16 | 1.39076194003072e-15 |
| *Rothia_mucilaginosa* | study_condition | Unhealthy | 0.378794661105272 | 0.047611232793215 | 1.87015632564344e-15 | 2.85520049716555e-15 |
| *Megamonas_funiformis* | study_condition | Unhealthy | 0.435409726624394 | 0.0569507090743467 | 2.17635164183157e-14 | 3.29750248762359e-14 |
| *Actinomyces_oris* | study_condition | Unhealthy | 0.318388063812186 | 0.0420978966012378 | 4.10593466661181e-14 | 6.17433784452904e-14 |
| *Phascolarctobacterium_succinatutens* | study_condition | Unhealthy | -0.82995 | 0.110218164021259 | 5.28386826280308e-14 | 7.88637054149714e-14 |
| *Bifidobacterium_animalis* | study_condition | Unhealthy | -0.36423 | 0.0492496766611948 | 1.46324827841999e-13 | 2.16777522728887e-13 |
| *Parabacteroides_merdae* | study_condition | Unhealthy | -0.9075 | 0.123092433888676 | 1.73958267993556e-13 | 2.55820982343464e-13 |
| *Clostridium_sp_CAG_242* | study_condition | Unhealthy | 0.34611109119524 | 0.047547875944446 | 3.48092671693517e-13 | 5.08164484224113e-13 |
| *Streptococcus_australis* | study_condition | Unhealthy | 0.272050155201819 | 0.0382411537914918 | 1.16443552966347e-12 | 1.68758772414995e-12 |
| *Lactococcus_lactis* | study_condition | Unhealthy | -0.3764 | 0.0534008583846015 | 1.86698669983893e-12 | 2.68631179832939e-12 |
| *Coprobacillus_cateniformis* | study_condition | Unhealthy | 0.304417783698611 | 0.0436509459550376 | 3.17783003354551e-12 | 4.5397571907793e-12 |
| *Eubacterium_sp_CAG_180* | study_condition | Unhealthy | -0.8578 | 0.127206307607431 | 1.58941603189562e-11 | 2.25449082538386e-11 |
| *Ruminococcaceae_bacterium_D16* | study_condition | Unhealthy | 0.271922057712988 | 0.0405180816334448 | 1.98252005249642e-11 | 2.79228176407947e-11 |
| *Bacteroides_massiliensis* | study_condition | Unhealthy | -0.60925 | 0.0908955259894206 | 2.09980416293805e-11 | 2.93678903907419e-11 |
| *Ruminococcaceae_bacterium_D5* | study_condition | Unhealthy | -0.26912 | 0.0413175334337871 | 7.52205502519694e-11 | 1.04472986461069e-10 |
| *Gordonibacter_pamelaeae* | study_condition | Unhealthy | -0.46285 | 0.074343688037185 | 4.88393285635563e-10 | 6.73645911221466e-10 |
| *Bacteroides_stercoris* | study_condition | Unhealthy | 0.738468058506629 | 0.120527626350054 | 9.12331432803561e-10 | 1.24976908603228e-09 |
| *Haemophilus_sp_HMSC71H05* | study_condition | Unhealthy | -0.25705 | 0.0424022861462519 | 1.36725499412244e-09 | 1.86021087635706e-09 |
| *Bacteroides_nordii* | study_condition | Unhealthy | 0.278373145925325 | 0.0465483150779102 | 2.26471390746986e-09 | 3.06042419928359e-09 |
| *Clostridium_sp_CAG_58* | study_condition | Unhealthy | -0.39876 | 0.0681865324914824 | 5.051686836763e-09 | 6.78078770035302e-09 |
| *Bacteroides_uniformis* | study_condition | Unhealthy | -0.43156 | 0.0806130637659184 | 8.72152634694181e-08 | 1.16287017959224e-07 |
| *Bifidobacterium_bifidum* | study_condition | Unhealthy | -0.51855 | 0.0983530884627475 | 1.36118833031986e-07 | 1.80289845075479e-07 |
| *Anaeromassilibacillus_sp_An250* | study_condition | Unhealthy | -0.37932 | 0.0724511572152738 | 1.66120510827486e-07 | 2.18579619509851e-07 |
| *Clostridium_leptum* | study_condition | Unhealthy | -0.39016 | 0.0761914798412364 | 3.07091307435683e-07 | 4.0142654566756e-07 |
| *Parabacteroides_johnsonii* | study_condition | Unhealthy | 0.328271217815516 | 0.0650131278882391 | 4.47277483112477e-07 | 5.80879848198022e-07 |
| *Bacteroides_plebeius* | study_condition | Unhealthy | 0.450116562351217 | 0.0949913773348663 | 2.1677368461268e-06 | 2.79707980145394e-06 |
| *Streptococcus_salivarius* | study_condition | Unhealthy | -0.32675 | 0.0752741055069731 | 1.42665473056306e-05 | 1.8290445263629e-05 |
| *Butyricimonas_synergistica* | study_condition | Unhealthy | -0.29866 | 0.069882672738859 | 1.93162091415792e-05 | 2.46066358491455e-05 |
| *Allisonella_histaminiformans* | study_condition | Unhealthy | 0.212798863000101 | 0.0502434519512652 | 2.29201747157906e-05 | 2.90128793870768e-05 |
| *Bacteroides_clarus* | study_condition | Unhealthy | -0.24582 | 0.060178350231575 | 4.42725961001603e-05 | 5.56888001259878e-05 |
| *Streptococcus_parasanguinis* | study_condition | Unhealthy | 0.306579243819897 | 0.0751443896649679 | 4.52342991183907e-05 | 5.65428738979884e-05 |
| *Bacteroides_coprocola* | study_condition | Unhealthy | 0.286532339899262 | 0.0721524245977806 | 7.17569271661252e-05 | 8.91390399579195e-05 |
| *Bacteroides_faecis* | study_condition | Unhealthy | -0.22104 | 0.0571870134709647 | 0.000111321455542438 | 0.000137433895731405 |
| *Veillonella_infantium* | study_condition | Unhealthy | 0.155570060081425 | 0.0408699551169634 | 0.000141383771057036 | 0.000173477019701885 |
| *Bacteroides_caccae* | study_condition | Unhealthy | -0.3859 | 0.107071726707596 | 0.00031401238112959 | 0.000382941928206817 |
| *Parasutterella_excrementihominis* | study_condition | Unhealthy | -0.31968 | 0.0897829935272095 | 0.000370948660991599 | 0.000449634740595878 |
| *Bacteroides_thetaiotaomicron* | study_condition | Unhealthy | 0.30757525237373 | 0.0888796647166674 | 0.00054012628065521 | 0.000650754555006277 |
| *Proteobacteria_bacterium_CAG_139* | study_condition | Unhealthy | -0.30183 | 0.0885255261213116 | 0.000652142105527197 | 0.000780006282888515 |
| *Alistipes_onderdonkii* | study_condition | Unhealthy | 0.224339453970921 | 0.0658234271070038 | 0.000655205277626353 | 0.000780006282888515 |
| *Actinomyces_odontolyticus* | study_condition | Unhealthy | 0.179976008352492 | 0.0528982378091143 | 0.000669494126326445 | 0.000792300741214728 |
| *Acidaminococcus_intestini* | study_condition | Unhealthy | 0.201074306054701 | 0.0605488072324704 | 0.000898987300688679 | 0.00105763211845727 |
| *Prevotella_stercorea* | study_condition | Unhealthy | -0.1973 | 0.0639883909070977 | 0.00204974591007494 | 0.00239736363751455 |
| *Bacteroides_eggerthii* | study_condition | Unhealthy | -0.28391 | 0.0935316995838638 | 0.00240510996014457 | 0.00279663948854019 |
| *Firmicutes_bacterium_CAG_145* | study_condition | Unhealthy | -0.16981 | 0.0586744073002048 | 0.0038064701229041 | 0.00440054349468682 |
| *Desulfovibrionaceae_bacterium* | study_condition | Unhealthy | -0.12267 | 0.0424434669106782 | 0.00385484395429009 | 0.00443085511987367 |
| *Butyricimonas_virosa* | study_condition | Unhealthy | -0.21846 | 0.0780085260885484 | 0.00510679424351473 | 0.00583633627830255 |
| *Monoglobus_pectinilyticus* | study_condition | Unhealthy | -0.14353 | 0.0549440083757267 | 0.00899988188891273 | 0.0102271385101281 |
| *Firmicutes_bacterium_CAG_94* | study_condition | Unhealthy | -0.13966 | 0.0554606393677151 | 0.0118017180010333 | 0.0133352745774388 |
| *Veillonella_dispar* | study_condition | Unhealthy | 0.113671437938158 | 0.0498858471429092 | 0.022699411593991 | 0.0255049568471809 |
| *Actinomyces_sp_ICM47* | study_condition | Unhealthy | 0.122662473994837 | 0.054969001791285 | 0.0256599954926521 | 0.0286703860253097 |
| *Haemophilus_parainfluenzae* | study_condition | Unhealthy | -0.16029 | 0.0739726367238912 | 0.0302539109442655 | 0.0335831873118133 |
| *Bacteroides_intestinalis* | study_condition | Unhealthy | -0.17976 | 0.083026763253341 | 0.030392784517191 | 0.0335831873118133 |
| *Ruthenibacterium_lactatiformans* | study_condition | Unhealthy | 0.148653182970511 | 0.0719587754123328 | 0.0388591275365859 | 0.0427023379522922 |

**Table S4.** Alpha diversity analysis of species.

| **shannon** | **Healthy** | **Unhealthy** | ***p*** |
| --- | --- | --- | --- |
| Mean | 2.94 | 0.513 | < 0.001 |
| Sd | 2.85 | 0.586 |  |
| **Simpson** | **Healthy** | **Unhealthy** | ***p*** |
| Mean | 0.882 | 0.105 | < 0.001 |
| Sd | 0.869 | 0.119 |  |
| **GMHI** | **Healthy** | **Unhealthy** | ***p*** |
| Mean | -0.218 | 2.06 | < 0.001 |
| Sd | -1.32 | 1.97 |  |

**Table S5.** Shannon diversity comparison across sex, age, and BMI groups.

| **MEF** | **Term** | ***p* value** |
| --- | --- | --- |
| Shannon | Sex | 1.4×10^-15^ |
| Shannon | age | 0.0036 |
| Shannon | BMI | >0.05 |

**Table S6.** Optimal k selection.

| **k** | **score** |
| --- | --- |
| 2 | 0.9614505 |
| 3 | 0.9957136 |
| 4 | 0.9770248 |
| 5 | 0.9699432 |
| 6 | 0.9719973 |
| 7 | 0.963481 |
| 8 | 0.9700988 |
| 9 | 0.9793461 |
| 10 | 0.9837168 |

**Table S7.** Dominant species analysis of MEFs.

| **MEF** | **Species** | **Abundance** |
| --- | --- | --- |
| MEF1 | *Bacteroides_uniformis* | 0.0957894301229443 |
| MEF1 | *Faecalibacterium_prausnitzii* | 0.0945197675063518 |
| MEF1 | *Bacteroides_vulgatus* | 0.0919544884284243 |
| MEF1 | *Ruminococcus_bromii* | 0.0560834553766634 |
| MEF1 | *Alistipes_putredinis* | 0.0539491127505171 |
| MEF1 | *Bacteroides_dorei* | 0.0403633880699211 |
| MEF1 | *Fusicatenibacter_saccharivorans* | 0.0342409123156365 |
| MEF1 | *Eubacterium_rectale* | 0.0309356337041971 |
| MEF1 | *Bacteroides_stercoris* | 0.029814505586593 |
| MEF1 | *Roseburia_faecis* | 0.0270123551366194 |
| MEF2 | *Prevotella_copri* | 0.500007698613589 |
| MEF2 | *Eubacterium_sp_CAG_180* | 0.0548460614947026 |
| MEF2 | *Butyrivibrio_crossotus* | 0.0428504414839251 |
| MEF2 | *Eubacterium_rectale* | 0.0348835015218603 |
| MEF2 | *Phascolarctobacterium_succinatutens* | 0.0304732128262593 |
| MEF2 | *Prevotella_stercorea* | 0.0253624651788135 |
| MEF2 | *Prevotella_sp_CAG_520* | 0.0218927132117758 |
| MEF2 | *Dialister_sp_CAG_357* | 0.0195112485746892 |
| MEF2 | *Prevotella_sp_CAG_279* | 0.0168416242143382 |
| MEF2 | *Oscillibacter_sp_57_20* | 0.0140641469390761 |
| MEF3 | *Bifidobacterium_adolescentis* | 0.0933026867188492 |
| MEF3 | *Collinsella_aerofaciens* | 0.0739180463134637 |
| MEF3 | *Anaerostipes_hadrus* | 0.0471588278076687 |
| MEF3 | *Bifidobacterium_longum* | 0.0463583827853518 |
| MEF3 | *Escherichia_coli* | 0.04382052878136 |
| MEF3 | *Eubacterium_rectale* | 0.0433667115652794 |
| MEF3 | *Dorea_longicatena* | 0.0374828563321651 |
| MEF3 | *Faecalibacterium_prausnitzii* | 0.0369426925150874 |
| MEF3 | *Ruminococcus_torques* | 0.0265561960035126 |
| MEF3 | *Blautia_wexlerae* | 0.0257665548178728 |

**Table S8.** lnCVR analysis of MEFs across groups.

| Factor | lnCVR | Significance |
| --- | --- | --- |
| MEF1 | 1.618604 | *p*<0.001 |
| MEF2 | 2.616933 | *p*<0.001 |
| MEF3 | 1.833149 | *p*<0.001 |

**Table S9.** Markov chain transition probabilities of MEFs.

| **From** | **To** | **Weight** |
| --- | --- | --- |
| MEF1 | MEF1 | 0.63 |
| MEF1 | MEF2 | 0.02 |
| MEF1 | MEF3 | 0.35 |
| MEF2 | MEF2 | 0.25 |
| MEF2 | MEF1 | 0.31 |
| MEF2 | MEF3 | 0.44 |
| MEF3 | MEF3 | 0.65 |
| MEF3 | MEF1 | 0.31 |
| MEF3 | MEF2 | 0.04 |

**Table S10.** Cosine similarity analysis of intergroup differences.

| **Subject_ID** | **cosine_mean** | **disease** |
| --- | --- | --- |
| 1 | 0.985841794351154 | control |
| 2 | 0.822638946736896 | Others |
| 3 | 0.978446602336815 | control |
| 4 | 0.997815225642563 | Others |
| 5 | 0.900286435466132 | Others |
| 6 | 0.835855905117305 | Others |
| 7 | 0.648104883774742 | Others |
| 8 | 0.561731774598758 | Others |
| 9 | 0.965367964618212 | Others |
| 10 | 0.94556853167904 | Others |
| 11 | 0.98467954712097 | control |
| 12 | 0.93197976746242 | Others |
| 13 | 0.92694075118718 | Others |
| 14 | 0.778011645987161 | Others |
| 15 | 0.704449866017496 | Others |
| 16 | 0.816714690255603 | Others |
| 17 | 0.977546345459692 | control |
| 18 | 0.975137929494107 | control |
| 19 | 0.95542525174342 | control |
| 20 | 0.773212704677306 | Others |
| 21 | 0.265008282176233 | Others |
| 22 | 0.978474974584042 | Others |
| 23 | 0.968400843878723 | control |
| 24 | 0.77003298920444 | Others |
| 25 | 0.984477281375518 | control |
| 26 | 0.77368151050998 | Others |
| 27 | 0.610426033806535 | Others |
| 28 | 0.882817279821338 | Others |
| 29 | 0.974948971769556 | control |
| 30 | 0.761527976752332 | Others |
| 31 | 0.959307271084095 | Others |
| 32 | 0.859971374056683 | Others |
| 33 | 0.921158859023043 | Others |
| 34 | 0.894649631156646 | Others |
| 35 | 0.973241990095747 | Others |
| 36 | 0.961277298960455 | Others |
| 37 | 0.853916982579619 | Others |
| 38 | 0.96197445223059 | Others |
| 39 | 0.984763988042754 | control |
| 40 | 0.982452365855551 | control |
| 41 | 0.97660866872558 | control |
| 42 | 0.98679805623415 | control |
| 43 | 0.966662182261255 | Others |
| 44 | 0.867208116499462 | Others |
| 45 | 0.89297261160904 | Others |
| 46 | 0.846600004327289 | Others |
| 47 | 0.859536979882043 | Others |
| 48 | 0.911668132111481 | Others |
| 49 | 0.945030395495865 | Others |
| 50 | 0.956537191294057 | control |
| 51 | 0.999413745706071 | control |
| 52 | 0.58471120788446 | Others |
| 53 | 0.831531735665729 | Others |
| 54 | 0.946471570560293 | control |
| 55 | 0.977374071877475 | control |
| 56 | 0.983905783329064 | control |
| 57 | 0.995920596990372 | Others |
| 58 | 0.722894003663001 | Others |
| 59 | 0.698692640827765 | Others |
| 60 | 0.975570173578838 | Others |
| 61 | 0.989694624911044 | control |
| 62 | 0.909502601846262 | Others |
| 63 | 0.724699880356202 | Others |
| 64 | 0.973742742604869 | control |
| 65 | 0.968969926434103 | control |
| 66 | 0.660503214472932 | Others |
| 67 | 0.879060292097627 | Others |
| 68 | 0.983223066861926 | control |
| 69 | 0.983001361774067 | control |
| 70 | 0.731921820006494 | Others |
| 71 | 0.926285104510134 | Others |
| 72 | 0.997685011109297 | control |
| 73 | 0.993676354591961 | control |
| 74 | 0.992545627000838 | control |
| 75 | 0.98525423778608 | control |
| 76 | 0.993177691898895 | control |
| 77 | 0.974046808431363 | control |
| 78 | 0.996707706477701 | control |
| 79 | 0.990893685976314 | control |
| 80 | 0.987919961719031 | control |
| 81 | 0.968066615099532 | control |
| 82 | 0.987137835929701 | control |
| 83 | 0.976891587498819 | control |
| 84 | 0.986302151252946 | control |
| 85 | 0.979902320286703 | control |
| 86 | 0.988643810139573 | control |
| 87 | 0.978779613833345 | control |
| 88 | 0.975032629358407 | control |
| 89 | 0.958878275316966 | control |
| 90 | 0.98981238833463 | control |
| 91 | 0.994392605660305 | control |
| 92 | 0.991250200066952 | control |
| 93 | 0.996960187578548 | control |
| 94 | 0.99463127273805 | control |
| 95 | 0.982861382539214 | control |
| 96 | 0.834439734448573 | Others |
| 97 | 0.834439734448573 | control |
| 98 | 0.997561616215323 | control |
| 99 | 0.999975765818842 | control |
| 100 | 0.991618744561539 | control |
| 101 | 0.995999112069113 | control |
| 102 | 0.991388392176959 | control |
| 103 | 0.959430601588822 | control |
| 104 | 0.781108160105691 | Others |
| 105 | 0.781108160105691 | control |
| 106 | 0.999997732282842 | control |
| 107 | 0.962948842568054 | Others |
| 108 | 0.868404208499839 | Others |
| 109 | 0.983552912445518 | Others |
| 110 | 0.736732098309173 | Others |
| 111 | 0.543915081556793 | Others |
| 112 | 0.640119740052361 | Others |
| 113 | 0.996332289838874 | Others |
| 114 | 0.645662432919623 | Others |
| 115 | 0.980791953841464 | Others |
| 116 | 0.86628951786893 | Others |
| 117 | 0.681248911678361 | Others |
| 118 | 0.748338806781508 | Others |
| 119 | 0.752626546660089 | Others |
| 120 | 0.792878020422384 | Others |
| 121 | 0.730761795685506 | Others |
| 122 | 0.632477287565917 | Others |
| 123 | 0.976939364585283 | Others |
| 124 | 0.975218943089443 | control |
| 125 | 0.831588185902544 | Others |
| 126 | 0.650323278889272 | Others |
| 127 | 0.987673894027563 | Others |
| 128 | 0.774807004006979 | Others |
| 129 | 0.936139738934697 | Others |
| 130 | 0.533467913267435 | Others |
| 131 | 0.782819458598039 | Others |
| 132 | 0.978869727220614 | Others |
| 133 | 0.715424557621404 | Others |
| 134 | 0.802065809394585 | Others |
| 135 | 0.938414463441083 | Others |
| 136 | 0.990953617794615 | control |
| 137 | 0.93911942852972 | control |
| 138 | 0.997345721161808 | control |
| 139 | 0.978846968289279 | control |
| 140 | 0.975842862633606 | control |
| 141 | 0.987063596308697 | control |
| 142 | 0.995198052505834 | control |
| 143 | 0.979702706287821 | control |
| 144 | 0.933017090351082 | control |
| 145 | 0.943254647170434 | control |
| 146 | 0.993747169012151 | control |
| 147 | 0.993099064755075 | control |
| 148 | 0.892037462013951 | Others |
| 149 | 0.892037462013951 | control |
| 150 | 0.996749872130968 | Others |
| 151 | 0.920215617692631 | Others |
| 152 | 0.920215617692631 | control |
| 153 | 0.996120218672529 | control |
| 154 | 0.939371469737842 | control |
| 155 | 0.894035490906457 | control |
| 156 | 0.894035490906457 | Others |
| 157 | 0.973523122323749 | control |
| 158 | 0.988180547719311 | control |
| 159 | 0.785898383622413 | Others |
| 160 | 0.785898383622413 | control |
| 161 | 0.989203205847591 | control |
| 162 | 0.93270268929396 | Others |
| 163 | 0.93270268929396 | control |
| 164 | 0.997807466331059 | control |
| 165 | 0.966852115787846 | control |
| 166 | 0.956090072634464 | control |
| 167 | 0.99654847573937 | control |
| 168 | 0.909700637789494 | Others |
| 169 | 0.909700637789494 | control |
| 170 | 0.992405158338583 | control |
| 171 | 0.882525213433038 | Others |
| 172 | 0.882525213433038 | control |
| 173 | 0.786042198364088 | Others |
| 174 | 0.786042198364088 | control |
| 175 | 0.780546126150945 | Others |
| 176 | 0.780546126150945 | control |
| 177 | 0.847544105486026 | Others |
| 178 | 0.847544105486026 | control |
| 179 | 0.614474322473746 | Others |
| 180 | 0.614474322473746 | control |
| 181 | 0.83504416747353 | Others |
| 182 | 0.755278620210529 | Others |
| 183 | 0.755278620210529 | control |
| 184 | 0.45575599784201 | Others |
| 185 | 0.856002586446238 | Others |
| 186 | 0.856002586446238 | control |
| 187 | 0.652790016334264 | Others |
| 188 | 0.652790016334264 | control |
| 189 | 0.798383608030771 | Others |
| 190 | 0.798383608030771 | control |
| 191 | 0.932827479791873 | Others |
| 192 | 0.754878840719006 | Others |
| 193 | 0.754878840719006 | control |
| 194 | 0.985810547631325 | Others |
| 195 | 0.898697983677126 | Others |
| 196 | 0.794636189525258 | Others |
| 197 | 0.794636189525258 | control |
| 198 | 0.826868532923621 | Others |
| 199 | 0.793504136707603 | Others |
| 200 | 0.503785940125966 | Others |
| 201 | 0.940773875833514 | Others |
| 202 | 0.723848088997363 | Others |
| 203 | 0.914504392870588 | Others |
| 204 | 0.521151513711083 | Others |
| 205 | 0.682798191878399 | Others |
| 206 | 0.985443965516374 | control |
| 207 | 0.977628848165431 | control |
| 208 | 0.988192385417532 | control |
| 209 | 0.989627963492711 | control |
| 210 | 0.993483310279629 | control |
| 211 | 0.994477864844051 | control |
| 212 | 0.992549073199965 | control |
| 213 | 0.737634291270285 | Others |
| 214 | 0.766560522472224 | Others |
| 215 | 0.739731796438666 | Others |
| 216 | 0.880545479451454 | Others |
| 217 | 0.978729233836778 | control |
| 218 | 0.949241080806694 | control |
| 219 | 0.984045343790405 | control |
| 220 | 0.976196064304319 | control |
| 221 | 0.870914996580379 | Others |
| 222 | 0.980705643254802 | control |
| 223 | 0.730703968692166 | Others |
| 224 | 0.976800430143273 | control |
| 225 | 0.930162931292068 | Others |
| 226 | 0.993216798498521 | control |
| 227 | 0.988025776426226 | control |
| 228 | 0.995949169581005 | control |
| 229 | 0.972622292158039 | control |
| 230 | 0.996298459209873 | control |
| 231 | 0.989735721035572 | control |
| 232 | 0.998302506996756 | control |
| 233 | 0.983846551755217 | control |
| 234 | 0.978227281892222 | control |
| 235 | 0.998105985985127 | control |
| 236 | 0.98416857238415 | control |
| 237 | 0.996196987472501 | control |
| 238 | 0.961145551817258 | control |
| 239 | 0.993967563533518 | control |
| 240 | 0.958795071050111 | control |
| 241 | 0.971310779575593 | control |
| 242 | 0.980729363879025 | control |
| 243 | 0.992854977826995 | control |
| 244 | 0.999495219038197 | control |
| 245 | 0.997427453386073 | control |
| 246 | 0.98967357198697 | control |
| 247 | 0.966351894568853 | control |
| 248 | 0.453889963941407 | Others |
| 249 | 0.976820120009441 | control |
| 250 | 0.983264506787212 | control |
| 251 | 0.996033444869168 | control |
| 252 | 0.997910345265113 | Others |
| 253 | 0.999633758530785 | control |
| 254 | 0.976777386111596 | control |
| 255 | 0.993331982066295 | control |
| 256 | 0.999352921099424 | control |
| 257 | 0.985877430214416 | control |
| 258 | 0.992249337391999 | control |
| 259 | 0.963276938124438 | control |
| 260 | 0.936314731725873 | control |
| 261 | 0.991549845978501 | control |
| 262 | 0.952097328996007 | control |
| 263 | 0.990471404067788 | control |
| 264 | 0.998040933438478 | control |
| 265 | 0.885493936182776 | control |
| 266 | 0.987648347717259 | control |
| 267 | 0.968299182552468 | Others |
| 268 | 0.989716098053688 | control |
| 269 | 0.939263072007938 | control |
| 270 | 0.992362051655029 | control |
| 271 | 0.989356204944087 | control |
| 272 | 0.996562213846171 | control |
| 273 | 0.950186402304518 | control |
| 274 | 0.996325401820846 | control |
| 275 | 0.991778062004524 | control |
| 276 | 0.998514899093627 | control |
| 277 | 0.995119088943648 | control |
| 278 | 0.915612239389465 | control |
| 279 | 0.998616875710342 | control |
| 280 | 0.944502656909831 | control |
| 281 | 0.991606666311369 | control |
| 282 | 0.985890395800911 | control |
| 283 | 0.985457316535003 | control |
| 284 | 0.963013114163777 | control |
| 285 | 0.386155195373669 | Others |
| 286 | 0.949057098982966 | control |
| 287 | 0.988995371980267 | control |
| 288 | 0.948271637238963 | control |
| 289 | 0.771243773989941 | control |
| 290 | 0.597984602896409 | Others |
| 291 | 0.868328447713287 | Others |
| 292 | 0.960935059296563 | control |
| 293 | 0.976453930015346 | control |
| 294 | 0.983356118224138 | control |
| 295 | 0.989993991553814 | control |
| 296 | 0.999988820593419 | control |
| 297 | 0.955299932348968 | control |
| 298 | 0.995252339171945 | control |
| 299 | 0.988447175608873 | control |
| 300 | 0.995753440584518 | control |
| 301 | 0.968413208451787 | control |
| 302 | 0.949228235461272 | control |
| 303 | 0.979835197867966 | control |
| 304 | 0.998468732556429 | control |
| 305 | 0.999959058807413 | control |
| 306 | 0.994665613028945 | control |
| 307 | 0.999959063366693 | control |
| 308 | 0.997211483403177 | control |
| 309 | 0.999671277427797 | control |
| 310 | 0.986365121493495 | control |
| 311 | 0.999878597322065 | control |
| 312 | 0.923518938947207 | control |
| 313 | 0.983958351914948 | control |
| 314 | 0.989136817658364 | control |
| 315 | 0.997389268972883 | control |
| 316 | 0.985927976875023 | control |
| 317 | 0.998174934003339 | control |
| 318 | 0.996470757422146 | control |
| 319 | 0.888745599840079 | Others |
| 320 | 0.888745599840079 | control |
| 321 | 0.960229485794506 | control |
| 322 | 0.833913913506555 | Others |
| 323 | 0.833913913506555 | control |
| 324 | 0.917631708713328 | Others |
| 325 | 0.917631708713328 | control |
| 326 | 0.988029224587585 | control |
| 327 | 0.974965406898435 | control |
| 328 | 0.887136758324794 | Others |
| 329 | 0.887136758324794 | control |
| 330 | 0.842539041904702 | Others |
| 331 | 0.842539041904702 | control |
| 332 | 0.992506525651173 | control |
| 333 | 0.782985424088274 | Others |
| 334 | 0.782985424088274 | control |
| 335 | 0.649564589492377 | Others |
| 336 | 0.649564589492377 | control |
| 337 | 0.812662435628365 | Others |
| 338 | 0.812662435628365 | control |
| 339 | 0.858442446914122 | Others |
| 340 | 0.858442446914122 | control |
| 341 | 0.861973694414283 | Others |
| 342 | 0.861973694414283 | control |
| 343 | 0.986307019668698 | Others |
| 344 | 0.830227512329876 | Others |
| 345 | 0.830227512329876 | control |
| 346 | 0.914663450284629 | Others |
| 347 | 0.914663450284629 | control |
| 348 | 0.742149918708283 | Others |
| 349 | 0.742149918708283 | control |
| 350 | 0.803864788058894 | Others |
| 351 | 0.803864788058894 | control |
| 352 | 0.834857311346404 | Others |
| 353 | 0.834857311346404 | control |
| 354 | 0.984328389197096 | control |
| 355 | 0.783415738294311 | control |
| 356 | 0.783415738294311 | Others |
| 357 | 0.98780179769416 | control |
| 358 | 0.980592633211783 | control |
| 359 | 0.985465798272288 | control |
| 360 | 0.994526759428423 | control |
| 361 | 0.986421746311367 | control |
| 362 | 0.860332162154761 | Others |
| 363 | 0.860332162154761 | control |
| 364 | 0.85617804235184 | Others |
| 365 | 0.85617804235184 | control |
| 366 | 0.888951793614433 | Others |
| 367 | 0.92812976167782 | Others |
| 368 | 0.695267798369272 | Others |
| 369 | 0.695267798369272 | control |
| 370 | 0.890512025949946 | Others |
| 371 | 0.998316749684003 | control |
| 372 | 0.851885333630205 | Others |
| 373 | 0.65374244506424 | Others |
| 374 | 0.999057184827014 | control |
| 375 | 0.971944335702285 | control |
| 376 | 0.991096452708418 | control |
| 377 | 0.576097203393659 | Others |
| 378 | 0.576097203393659 | control |
| 379 | 0.978425507777743 | control |
| 380 | 0.985025808420031 | Others |
| 381 | 0.988949019983048 | control |
| 382 | 0.86784874610501 | control |
| 383 | 0.86784874610501 | Others |
| 384 | 0.776716313682209 | Others |
| 385 | 0.776716313682209 | control |
| 386 | 0.945736155877953 | Others |
| 387 | 0.731460794768614 | Others |
| 388 | 0.731460794768614 | control |
| 389 | 0.839112349636512 | Others |
| 390 | 0.839112349636512 | control |
| 391 | 0.855503703441381 | Others |
| 392 | 0.855503703441381 | control |
| 393 | 0.980741074160502 | Others |
| 394 | 0.935520547330318 | Others |
| 395 | 0.991889970458268 | Others |
| 396 | 0.853483597249777 | Others |
| 397 | 0.853483597249777 | control |
| 398 | 0.950839197130351 | Others |
| 399 | 0.993952862545265 | control |
| 400 | 0.555816155238181 | Others |
| 401 | 0.993883011488489 | control |
| 402 | 0.993735964248452 | Others |
| 403 | 0.717675727116326 | Others |
| 404 | 0.717675727116326 | control |
| 405 | 0.931099702950608 | control |
| 406 | 0.931099702950608 | Others |
| 407 | 0.762784057186126 | Others |
| 408 | 0.762784057186126 | control |
| 409 | 0.841450719790895 | Others |
| 410 | 0.841450719790895 | control |
| 411 | 0.897085400456617 | Others |
| 412 | 0.897085400456617 | control |
| 413 | 0.885214473459282 | Others |
| 414 | 0.885214473459282 | control |
| 415 | 0.994912864517176 | control |
| 416 | 0.995528962723004 | control |
| 417 | 0.995541426922263 | Others |
| 418 | 0.961379846453437 | Others |
| 419 | 0.804602927636523 | Others |
| 420 | 0.804602927636523 | control |
| 421 | 0.875067129032413 | control |
| 422 | 0.875067129032413 | Others |
| 423 | 0.98725217978683 | control |
| 424 | 0.988223773518773 | control |
| 425 | 0.983129248760083 | control |
| 426 | 0.853029519135548 | Others |
| 427 | 0.853029519135548 | control |
| 428 | 0.747572745472363 | Others |
| 429 | 0.747572745472363 | control |
| 430 | 0.900618007750294 | Others |
| 431 | 0.900618007750294 | control |
| 432 | 0.792472562978396 | Others |
| 433 | 0.792472562978396 | control |
| 434 | 0.802862612501642 | Others |
| 435 | 0.998515742982485 | Others |
| 436 | 0.88707937814118 | Others |
| 437 | 0.88707937814118 | control |
| 438 | 0.656980446683855 | Others |
| 439 | 0.656980446683855 | control |
| 440 | 0.96836715004738 | control |
| 441 | 0.823750761349268 | Others |
| 442 | 0.823750761349268 | control |
| 443 | 0.812952995646771 | Others |
| 444 | 0.812952995646771 | control |
| 445 | 0.99306454426196 | control |
| 446 | 0.840622817412303 | Others |
| 447 | 0.840622817412303 | control |
| 448 | 0.991065644793756 | control |
| 449 | 0.977715426839242 | control |
| 450 | 0.985876127353063 | control |
| 451 | 0.747190974466671 | Others |
| 452 | 0.747190974466671 | control |
| 453 | 0.830004708050865 | control |
| 454 | 0.830004708050865 | Others |
| 455 | 0.995289318501668 | control |
| 456 | 0.98452579785177 | control |
| 457 | 0.989278576705307 | control |
| 458 | 0.986089013279203 | control |
| 459 | 0.898724938226966 | Others |
| 460 | 0.898724938226966 | control |
| 461 | 0.718993821226861 | Others |
| 462 | 0.978439107512065 | control |
| 463 | 0.991176759754097 | control |
| 464 | 0.918497384547506 | Others |
| 465 | 0.918497384547506 | control |
| 466 | 0.911012565381897 | Others |
| 467 | 0.911012565381897 | control |
| 468 | 0.970027832839934 | control |
| 469 | 0.986296535242739 | control |
| 470 | 0.855895037349137 | Others |
| 471 | 0.855895037349137 | control |
| 472 | 0.868309402256212 | Others |
| 473 | 0.818648113732164 | Others |
| 474 | 0.818648113732164 | control |
| 475 | 0.961131855785496 | control |
| 476 | 0.996361157643626 | control |
| 477 | 0.977253652837416 | control |
| 478 | 0.845201686943352 | Others |
| 479 | 0.845201686943352 | control |
| 480 | 0.980609814883359 | control |
| 481 | 0.985088363861964 | control |
| 482 | 0.723844242681136 | Others |
| 483 | 0.723844242681136 | control |
| 484 | 0.85018289279303 | Others |
| 485 | 0.85018289279303 | control |
| 486 | 0.851686103863636 | Others |
| 487 | 0.851686103863636 | control |
| 488 | 0.816020181995586 | Others |
| 489 | 0.816020181995586 | control |
| 490 | 0.995047248969453 | control |
| 491 | 0.848348393548044 | Others |
| 492 | 0.848348393548044 | control |
| 493 | 0.992564024428194 | control |
| 494 | 0.994004698057624 | Others |
| 495 | 0.96260850654534 | Others |
| 496 | 0.983446056520648 | control |
| 497 | 0.898932524680856 | Others |
| 498 | 0.898932524680856 | control |

**Table S11.** Linear mixed-effects model analysis of MEFs across diagnostic groups.

| **MEF** | **Variable** | **Beta** | **SE** | **p_value** | **Signif** |
| --- | --- | --- | --- | --- | --- |
| MEF1 | (Intercept) | -0.032703752 | 0.098329089 | 0.739493809 | ns |
| MEF1 | GroupMDD | 0.091271316 | 0.01468351 | 6.86387E-10 | *** |
| MEF1 | GroupBD | 0.042235639 | 0.019396805 | 0.029626365 | * |
| MEF1 | GroupSZ | 0.003956683 | 0.024294035 | 0.870648932 | ns |
| MEF2 | (Intercept) | 0.266288716 | 0.037980538 | 3.4842E-06 | *** |
| MEF2 | GroupMDD | -0.184276094 | 0.004044268 | 2.2947E-271 | *** |
| MEF2 | GroupBD | -0.236324662 | 0.006055628 | 4.8161E-221 | *** |
| MEF2 | GroupSZ | -0.247281969 | 0.007105621 | 5.5888E-188 | *** |
| MEF3 | (Intercept) | -0.21187162 | 0.104121482 | 0.042506519 | * |
| MEF3 | GroupMDD | 0.093125226 | 0.01517805 | 1.12841E-09 | *** |
| MEF3 | GroupBD | 0.203924394 | 0.022424181 | 5.40429E-19 | *** |
| MEF3 | GroupSZ | 0.238434817 | 0.026469517 | 8.67099E-19 | *** |

**Table S12.** MEFs expression analysis in major psychiatric disorders.

| **Group** | **MEF1** | **MEF2** | **MEF3** | **SD1** | **SD2** | **SD3** |
| --- | --- | --- | --- | --- | --- | --- |
| Healthy | 0.3530341 | 0.284570746 | 0.3623952 | 0.05241661 | 0.05308908 | 0.05608205 |
| MDD | 0.4611893 | 0.007415239 | 0.5313955 | 0.26347133 | 0.01338499 | 0.26149601 |
| BD | 0.4246806 | 0.026277763 | 0.5490416 | 0.29236016 | 0.11737198 | 0.29708277 |
| SZ | 0.3742824 | 0.015585858 | 0.6101317 | 0.26648095 | 0.03430296 | 0.26913869 |

**Table S13.** Cognitive impairment in MDD and SZ.

| **Measure** | **MDD_d** | **SZ_d** |
| --- | --- | --- |
| SoP.AGET | -0.7 | -0.58 |
| AV.AGET | -1.39 | -0.73 |
| WM.AGET | -1.17 | -0.54 |
| Vrbl.Lrng.AGET | -0.73 | -0.72 |
| Vis.Lrng.AGET | -0.81 | -0.4 |

**Table S14.** Associations between MEFs and clinical symptom severity.

| **MEF** | **Clinical** | **Correlation** | **signif_label** |
| --- | --- | --- | --- |
| MEF1 | HAMD_1 | -0.150099986 | * |
| MEF2 | HAMD_1 | 0.0203991182115673 |  |
| MEF3 | HAMD_1 | 0.147696398532068 | * |
| MEF1 | HAMD_2 | 0.0326193455326711 |  |
| MEF2 | HAMD_2 | -0.103794491 |  |
| MEF3 | HAMD_2 | -0.032615289 |  |
| MEF1 | HAMD_3 | 0.0427652547176084 |  |
| MEF2 | HAMD_3 | -0.086581189 |  |
| MEF3 | HAMD_3 | -0.040396724 |  |
| MEF1 | HAMD_4 | 0.0475085093384204 |  |
| MEF2 | HAMD_4 | -0.026350978 |  |
| MEF3 | HAMD_4 | -0.049090304 |  |
| MEF1 | HAMD_5 | 0.0759671833157131 |  |
| MEF2 | HAMD_5 | 0.0245018686098145 |  |
| MEF3 | HAMD_5 | -0.078324582 |  |
| MEF1 | HAMD_6 | 0.0352989124512686 |  |
| MEF2 | HAMD_6 | -0.008388347 |  |
| MEF3 | HAMD_6 | -0.038272208 |  |
| MEF1 | HAMD_7 | -0.013724607 |  |
| MEF2 | HAMD_7 | -0.051962055 |  |
| MEF3 | HAMD_7 | 0.0136650507437931 |  |
| MEF1 | HAMD_8 | 0.0049612208458528 |  |
| MEF2 | HAMD_8 | 0.0353099120856699 |  |
| MEF3 | HAMD_8 | -0.006312866 |  |
| MEF1 | HAMD_9 | -0.007127827 |  |
| MEF2 | HAMD_9 | 0.218236783486817 | ** |
| MEF3 | HAMD_9 | -0.006723049 |  |
| MEF1 | HAMD_10 | -0.000618323 |  |
| MEF2 | HAMD_10 | 0.0644043346337031 |  |
| MEF3 | HAMD_10 | -0.007636242 |  |
| MEF1 | HAMD_11 | 0.0730973744648268 |  |
| MEF2 | HAMD_11 | -0.085340925 |  |
| MEF3 | HAMD_11 | -0.073803627 |  |
| MEF1 | HAMD_12 | -0.085062634 |  |
| MEF2 | HAMD_12 | -0.042063014 |  |
| MEF3 | HAMD_12 | 0.086600917555735 |  |
| MEF1 | HAMD_13 | -0.027662627 |  |
| MEF2 | HAMD_13 | -0.054070954 |  |
| MEF3 | HAMD_13 | 0.0308547279783343 |  |
| MEF1 | HAMD_14 | 0.0548144006202889 |  |
| MEF2 | HAMD_14 | -0.10640987 |  |
| MEF3 | HAMD_14 | -0.051650252 |  |
| MEF1 | HAMD_15 | 0.0942597537336336 |  |
| MEF2 | HAMD_15 | -0.096496388 |  |
| MEF3 | HAMD_15 | -0.101656511 |  |
| MEF1 | HAMD_16 | -0.044041873 |  |
| MEF2 | HAMD_16 | 0.019147456957097 |  |
| MEF3 | HAMD_16 | 0.0394466838817509 |  |
| MEF1 | HAMD_17 | -0.05312739 |  |
| MEF2 | HAMD_17 | 0.0108101156727645 |  |
| MEF3 | HAMD_17 | 0.055526025171943 |  |
| MEF1 | HAMD-depression | 0.0291187760577076 |  |
| MEF2 | HAMD-depression | -0.051130181 |  |
| MEF3 | HAMD-depression | -0.034106546 |  |
| MEF1 | HAMA-anxiety | 0.066482338409902 |  |
| MEF2 | HAMA-anxiety | -0.023337328 |  |
| MEF3 | HAMA-anxiety | -0.070088052 |  |
